# The circadian clock component BMAL1 regulates SARS-CoV-2 entry and replication in lung epithelial cells

**DOI:** 10.1016/j.isci.2021.103144

**Published:** 2021-09-16

**Authors:** Xiaodong Zhuang, Senko Tsukuda, Florian Wrensch, Peter A.C. Wing, Mirjam Schilling, James M. Harris, Helene Borrmann, Sophie B. Morgan, Jennifer L. Cane, Laurent Mailly, Nazia Thakur, Carina Conceicao, Harshmeena Sanghani, Laura Heydmann, Charlotte Bach, Anna Ashton, Steven Walsh, Tiong Kit Tan, Lisa Schimanski, Kuan-Ying A. Huang, Catherine Schuster, Koichi Watashi, Timothy S.C. Hinks, Aarti Jagannath, Sridhar R. Vausdevan, Dalan Bailey, Thomas F. Baumert, Jane A. McKeating

**Affiliations:** 1 Nuffield Department of Medicine, University of Oxford, Oxford, UK; 2 Université de Strasbourg, Strasbourg, France and INSERM, U1110, Institut de Recherche sur les Maladies Virales et Hépatiques, Strasbourg, France; 3 Chinese Academy of Medical Sciences (CAMS) Oxford Institute (COI), University of Oxford, Oxford, UK; 4 Respiratory Medicine Unit and National Institute for Health Research Oxford Biomedical Research Centre, Nuffield Department of Medicine, Experimental Medicine, University of Oxford, UK; 5 The Pirbright Institute, Ash Road, Pirbright, Woking, Surrey, UK; 6 Nuffield Department of Clinical Neurosciences, University of Oxford, Oxford, UK; 7 Department of Pharmacology, University of Oxford, Oxford, UK; 8 MRC Human Immunology Unit, MRC Weatherall Institute, John Radcliffe Hospital, Oxford 17 OX3 9DS, UK; 9 Research Center for Emerging Viral Infections, College of Medicine, Chang Gung University and Division of Pediatric Infectious Diseases, Department of Pediatrics, Chang Gung Memorial Hospital, Taoyuan, Taiwan; 10 Department of Virology II, National Institute of Infectious Diseases, Tokyo 162-8640, Japan; 11 Department of Applied Biological Science, Tokyo University of Science, Noda 278-8510, Japan; 12 Pole Hépato-digestif, IHU, Hopitaux Universitaires de Strasbourg, Strasbourg, France

**Keywords:** Molecular biology, Microbiology, Virology, Transcriptomics

## Abstract

The coronavirus disease 2019 pandemic, caused by severe acute respiratory syndrome coronavirus 2 (SARS-CoV-2) coronavirus, is a global health issue with unprecedented challenges for public health. SARS-CoV-2 primarily infects cells of the respiratory tract via spike glycoprotein binding to angiotensin-converting enzyme (ACE2). Circadian rhythms coordinate an organism's response to its environment and can regulate host susceptibility to virus infection. We demonstrate that silencing the circadian regulator *Bmal1* or treating lung epithelial cells with the REV-ERB agonist SR9009 reduces ACE2 expression and inhibits SARS-CoV-2 entry and replication. Importantly, treating infected cells with SR9009 limits SARS-CoV-2 replication and secretion of infectious particles, showing that post-entry steps in the viral life cycle are influenced by the circadian system. Transcriptome analysis revealed that *Bmal1* silencing induced interferon-stimulated gene transcripts in Calu-3 lung epithelial cells, providing a mechanism for the circadian pathway to limit SARS-CoV-2 infection. Our study highlights alternative approaches to understand and improve therapeutic targeting of SARS-CoV-2.

## Highlights

• BMAL1 regulates ACE2-dependent SARS-CoV-2 entry
• REV-ERB agonist reduces ACE2-dependent cell-cell fusion
• Genetic or pharmacological targeting of BMAL1 reduces SARS-CoV-2 replication
• BMAL1 regulates interferon-stimulated gene expression

## Graphical abstract

**Figure:**
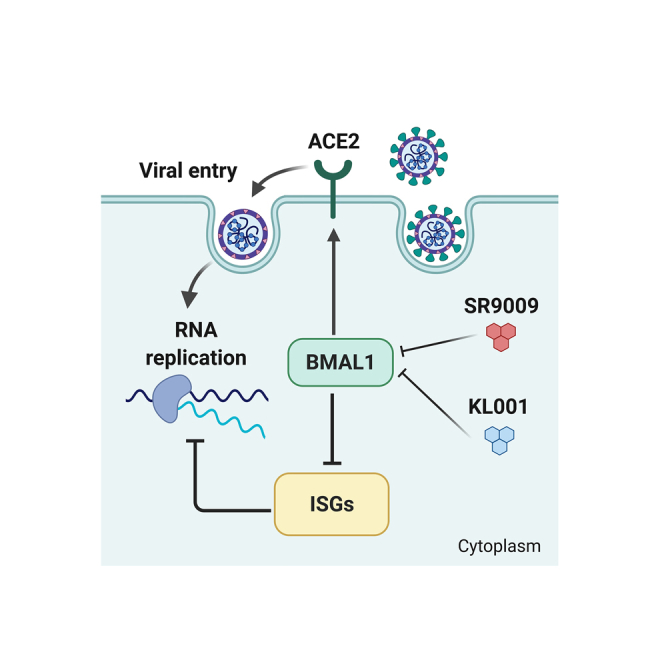

## Introduction

Severe acute respiratory syndrome coronavirus 2 (SARS-CoV-2) primarily targets the respiratory tract (Hou et al., 2020) resulting in pneumonia and an acute respiratory distress syndrome, especially in the elderly and individuals with comorbidities (Guan et al., 2020). There is an urgent need for prophylactic measures or early treatments that reduce disease severity and fatalities in vulnerable groups. Infection is mediated by the spike protein binding human angiotensin-converting enzyme (ACE2); cleavage of spike by the transmembrane protease serine 2 (TMPRSS2) triggers fusion of the viral and cell membranes (Hoffmann et al., 2020b; Wan et al., 2020). ACE2 is highly expressed in epithelia of the respiratory tract as well as those of the kidney and intestine (Hamming et al., 2004; Tipnis et al., 2000; Zhao et al., 2020). Reports showing evidence of multi-organ involvement in severe coronavirus disease 2019 (COVID-19) (Gavriatopoulou et al., 2020), including the gastrointestinal tract (Trottein and Sokol, 2020) and central nervous system (Marshall, 2020), demonstrate the complexities of this disease.

Since the initial outbreak in the Wuhan Province of China in 2019, several variants of concern (VOCs) have been documented that show a fitness advantage in terms of their ability to transmit within the community (Caly et al., 2020; Davies et al., 2021; Kidd et al., 2021; Volz et al., 2021). Many of the VOC encode mutations in the spike (S) protein (Rees-Spear et al., 2021) can impact viral entry and immunoevasion (Kissler et al., 2021; Washington et al., 2021), highlighting the urgent need for therapies that will be effective against all variants.

Circadian signaling exists in nearly every cell and is primarily controlled by a series of transcription/translation feedback loops. The transcriptional activators BMAL1 and CLOCK regulate thousands of transcripts including their own repressors, REV-ERBα and REV-ERBβ, that provide a negative feedback loop to control gene expression. One of the defining hallmarks of circadian regulated processes is the impact of the time of day on infection outcome. In diverse models of viral or bacterial infections, the disruption of the clock incurred by the loss of *Bmal1* in key cell types increased disease severity (Early et al., 2018; Ehlers et al., 2018; Gibbs et al., 2014; Pariollaud et al., 2018; Scheiermann et al., 2012; Sengupta et al., 2019; Sutton et al., 2017; Zhang et al., 2019). Human lung diseases frequently show time-of-day variation in symptom severity and respiratory function (Scheiermann et al., 2018), and BMAL1 is recognized to play a key role in regulating pulmonary inflammation (Ince et al., 2019). Influenza A infection of arrhythmic mice, lacking *Bmal1*, is associated with a higher viral burden in the lung (Edgar et al., 2016) and elevated inflammatory responses (Ehlers et al., 2018; Sengupta et al., 2019). We (Sengupta et al., 2021) and others (Maiese, 2020; Ray and Reddy, 2020) hypothesized a potential role for the circadian clock to regulate SARS-CoV-2 replication and COVID-19 severity. This study highlights the importance of BMAL1 and REV-ERB in regulating multiple aspects of SARS-CoV-2 infection and raises the potential use of circadian-modifying agents in the prevention and/or treatment of COVID-19.

## Results

### BMAL1 regulation of ACE2-dependent SARS-CoV-2 entry

**Figure 1:**
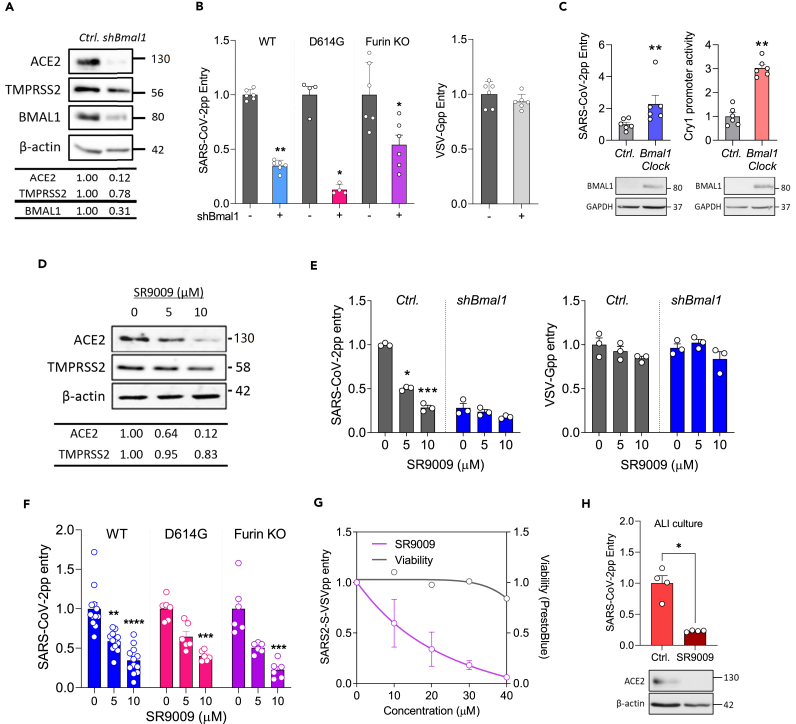
BMAL1 regulation of ACE2-dependent SARS-CoV-2 entry (A) Calu-3 cells were transduced with lentivirus encoding sh*Bmal1* or control and ACE2, TMPRSS2, BMAL1, and β-actin expression assessed by western blotting. (B) Control or sh*Bmal1* treated cells were infected with lentiviral pseudotypes expressing wild-type (WT) or mutant (D614G or Furin KO) spike variants or with VSV-Gpp. Viral entry (luciferase activity) was measured 24 h later, and data are expressed relative to control cells (Ctrl) (mean ± S.E.M., n = 4–6, Mann-Whitney test). (C) Calu-3 cells transfected with BMAL1/CLOCK expression plasmids were infected with SARS-CoV-2pp or co-transfected with Cry1 promoter luciferase reporter. Then, 48 h after transfection, cells were lysed and viral entry or promoter activity determined by quantifying luciferase activity, and data are expressed relative to control treatment (mean ± SEM, n = 6, Mann-Whitney test). Cell lysates were probed with anti-Flag to assess exogenous BMAL1 expression together with anti-GAPDH by western blotting. (D) Calu-3 cells were treated with SR9009 (5 or 10 μM) for 24 h and assessed for ACE2 and TMPRSS2 expression together with housekeeping β-actin. (E) Control or sh*Bmal1* silenced Calu-3 cells were treated with SR9009 for 24 h followed by infection with SARS-CoV-2pp or VSV-Gpp. After 24 h, viral entry was assessed by measuring luciferase activity and expressed relative to untreated cells (mean ± S.E.M., n = 3, Kruskal-Wallis ANOVA with Dunn's test). (F) Control or SR9009-treated Calu-3 cells were infected with SARS-CoV-2 WT, D614G, or furin mutant pp. Viral entry was expressed relative to untreated cells (mean ± S.E.M., n = 4–6, Kruskal-Wallis ANOVA with Dunn's test). (G) Control or SR9009-treated Calu-3 cells were infected with SARS2-S-VSVpp, and 24 h later, luciferase activity was measured, and data are expressed relative to untreated cells (mean ± S.E.M., n = 3). Cell viability was determined using PrestoBlue cell viability assay. (H) Differentiated air-liquid interface cultures of proximal airway epithelial cells were treated with SR9009 (20 μM) for 24 h prior to SARS-CoV-2pp infection. Viral entry was measured as luciferase activity, and data are presented relative to untreated control cells (mean ± S.E.M., n = 4, Mann-Whitney test). ACE2 expression together with housekeeping β-actin. See related Figures S1, S2 and S3 ∗p < 0.05; ∗∗p < 0.01; ∗∗∗p < 0.001; ∗∗∗∗p < 0.0001.

Since ACE2 and TMPRSS2 mediate SARS-CoV-2 internalization (Hoffmann et al., 2020b; Wan et al., 2020), the first step of the viral life cycle, we explored the role of circadian pathways in regulating these entry factors. We selected to use human lung Calu-3 epithelial cells that have intact innate sensing pathways (Cao et al., 2021; Li et al., 2021) and are widely used to study SARS-CoV-2 replication (Chu et al., 2020; Hoffmann et al., 2020b). Silencing *Bmal1*, the major circadian transcriptional activator, reduced ACE2 expression with a negligible effect on TMPRSS2 (Figure 1A). Since the classical model of circadian regulation is one of the transcriptional controls, we quantified *Ace2* and *Tmprss2* transcripts in both the lung and liver harvested from light/dark entrained mice and observed limited evidence for a circadian pattern of expression (Figure S1). BMAL1 and REV-ERB regulate gene expression by binding E-box or ROR response elements (ROREs), respectively, in the promoter and enhancer regions of their target genes (Harding and Lazar, 1993). The human ACE2 promoter encodes putative binding sites for BMAL1/CLOCK and the circadian repressor REV-ERB; however, we did not observe any binding of these factors by chromatin immunoprecipitation-qPCR in Calu-3 cells (Figure S2). Furthermore, there was no evidence for a rhythmic pattern of *Ace2* transcripts in Calu-3 or in published diurnal/circadian data sets from baboons (Mure et al., 2018) or mice (Zhang et al., 2019), suggesting a post-transcriptional regulation of ACE2.

Lentiviruses can incorporate exogenous viral glycoproteins, and the resulting pseudoparticles (pps) undergo a single cycle of infection that enables the study of receptor-specific internalization pathways. To assess the impact of BMAL1 on SARS-CoV-2pp entry, we infected the silenced Calu-3 cells and showed a significant reduction in pp infection, whereas VSV-Gpp infection was unaffected (Figure 1B). During the COVID-19 pandemic, several spike variants have emerged: some conferring a fitness advantage to viral entry. D614G is prevalent in many countries, consistent with a reported advantage for infecting cells of the upper respiratory tract (Korber et al., 2020). SARS-CoV-2 spike has a unique furin cleavage site that mediates membrane fusion, and deletion of this motif is associated with reduced pathogenesis (Johnson et al., 2021). Importantly, pp containing either spike variants showed reduced infection of *Bmal1*-silenced Calu-3 cells (Figure 1B). To further explore a regulatory role for BMAL1 in SARS-CoV-2 entry, we overexpressed BMAL1 along with CLOCK in Calu-3 cells and confirmed expression by western blotting, showing a significant increase in both SARS-CoV-2pp entry and promoter activity of the host target Cry1 (Figure 1C). In summary, these data show a role for BMAL1 to regulate ACE2 and SARS-CoV-2 entry.

The availability of a synthetic agonist (SR9009) that activates REV-ERB and modulates circadian pathways (Solt et al., 2012; Trump et al., 2013) prompted us to investigate its role in SARS-CoV-2 infection. Treating Calu-3 cells with SR9009 reduced BMAL1 promoter activity and protein expression, with no effect on cell viability (Figure S3). SR9009 treatment reduced ACE2 expression in a dose-dependent manner but had no effect on TMPRSS2 expression (Figure 1D). Furthermore, SARS-CoV-2pp infection was significantly reduced by SR9009 treatment in parental Calu-3 cells but not in sh*Bmal1* silenced cells, demonstrating a BMAL1 dependency (Figure 1E). In contrast, SR9009 treatment had no effect on VSV-Gpp infection (Figure 1E). SR9009 treatment inhibited the infection of pp bearing the D614G or furin-KO spike variants (Figure 1F). Using an independent pp system based on Vesicular Stomatitis Virus (VSV) (Hoffmann et al., 2020b), we show that SR9009 treatment of Calu-3 reduced particle infection (Figure 1G). To extend our observations to a more physiologically relevant system, we treated differentiated air-liquid interface (ALI) cultures of proximal airway epithelial cells with SR9009 and showed a significant reduction in SARS-CoV-2pp infection and ACE2 expression (Figure 1H).

### REV-ERB agonist reduces ACE2-dependent cell-cell fusion

**Figure 2:**
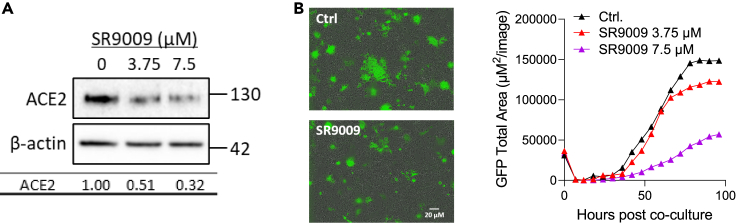
REV-ERB agonist reduces ACE2-dependent cell-cell fusion (A) SR9009-treated Huh-7 cells were assessed for ACE2 expression together with the housekeeping gene β-actin. Densitometric analysis quantified ACE2 in individual samples and were normalized to the β-actin loading control. Data are representative of three experiments. (B) Huh-7 target cells expressing a split rLuc-GFP reporter (8–11) were treated overnight with SR9009 at the indicated concentrations or with DMSO vehicle (Ctrl.) before culturing with spike-expressing HEK-293T cells with the split rLuc-GFP (1–7) and SR9009 added for the indicated times. A representative image of SARS-CoV-2 spike induced syncytia at 3 days post co-culture is shown (left image). GFP-positive syncytia were quantified every 4h using an IncuCyte real-time imaging platform (right image). Five fields of view were obtained per well at 10× magnification, and GFP expression was quantified by calculating the total GFP area using IncuCyte analysis software.

SARS-CoV-2 spike binding to ACE2 can induce formation of multicellular syncytia (Sanders et al., 2021; Xu et al., 2020), and we used a real-time assay to assess the effect of SR9009 on spike-dependent cell-cell fusion (Thakur et al., 2021). Initial experiments evaluated Calu-3 as target cells in this fusion assay, but due to their preferred growth in clusters, we obtained high inter- and intra-assay variability. In contrast, Huh-7 hepatoma cells, previously used to study SARS-CoV-1 infection (Gillim-Ross et al., 2004; Lambert et al., 2005; Nie et al., 2004), that express endogenous ACE2 and grow as a monolayer, supported SARS-CoV-2 spike cell-cell fusion. We confirmed that treating Huh-7 cells with SR9009 reduced ACE2 expression (Figure 2A) and spike-driven cell-cell fusion (Figure 2B). In summary, the REV-ERB agonist SR9009 represses ACE2 expression and limits SARS-CoV-2-mediated cell-cell fusion.

### Genetic or pharmacological targeting BMAL1 reduces SARS-CoV-2 replication

**Figure 3:**
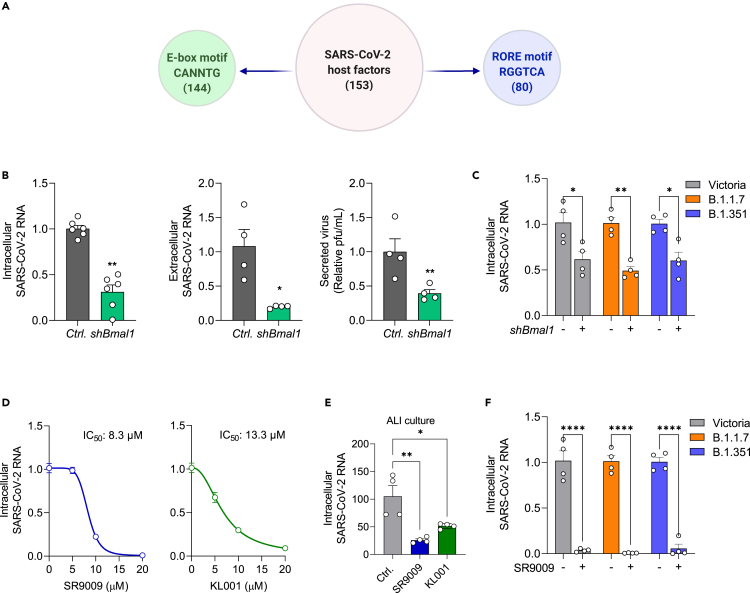
Genetic or pharmacological targeting of BMAL1 reduces SARS-CoV-2 replication (A) Circadian regulatory motifs in the promoter of proposed SARS-CoV-2 host factors. Sequence analysis of promoters of genes encoding potential SARS-CoV-2 host genes with HOMER (Hypergeometric Optimization of Motif EnRichment tool) identifies a canonical E-box motif “CANNTG” in 144 of the 153 genes and a ROR response element “RCGTCA” in 80. (B) *Bmal1* silencing reduces SARS-CoV-2 replication. Calu-3 cells were transduced with lentivirus encoding sh*Bmal1* or a control (Ctrl) followed by SARS-Cov-2 infection. Viral RNA was measured at 24 h post-infection (hpi) and expressed relative to Ctrl. Infectivity of the extracellular viral particles was assessed by plaque assay using Vero-TMPRSS2 cells and the mean infectious titers from Ctrl (1.15 × 10^6^ PFU/mL) and sh*Bmal1 (*4.5 × 10^5^ PFU/mL) noted and plotted relative to Ctrl (mean ± S.E.M., n = 4–6, Mann-Whitney test). (C) Calu-3 cells stably transduced with lentivirus encoding sh*Bmal1* or a control were infected with SARS-CoV-2 Victoria 01/20, B1.1.7 or B1.351 (MOI 0.003) and viral replication assessed 24hpi by measuring intracellular viral RNA and data expressed relative to the control (mean ± S.E.M., n = 4, Mann-Whitney test). (D) Calu-3 cells were treated with REV-ERB agonist SR9009 or the cryptochrome stabilizer KL001 for 24h and infected with SARS-Cov-2. Intracellular viral RNA was measured at 24hpi and expressed relative to the untreated control. Data are presented as mean ± S.E.M. from n = 4 independent biological replicates. (E) Differentiated air-liquid interface cultures of proximal airway epithelial cells were treated with SR9009 (20 μM) or KL001 (20 μM) for 24h followed by SARS-CoV-2 infection. Intracellular viral RNA was quantified at 24hpi and expressed relative to the untreated control. Data are presented as mean ± S.E.M., n = 4, Kruskal-Wallis ANOVA with Dunn's test. (F) Calu-3 cells were treated with SR9009 for 24h followed by infection with SARS-CoV-2 Victoria 01/20, B1.1.7 or B1.351 (MOI 0.003) and viral replication assessed at 24hpi by measuring intracellular viral RNA and data expressed relative to the untreated control (mean ± S.E.M., n = 4, Mann-Whitney test). ∗p < 0.05; ∗∗p < 0.01; ∗∗∗p < 0.001; ∗∗∗∗p < 0.0001. See related Figure S4.

A published genome-wide CRISPR screen identified 153 host factors with a potential role in SARS-CoV-2 infection (Daniloski et al., 2021). Bioinformatic analysis identified 144 canonical E-box motifs “CANNTG” and 80 ROREs “RGGTCA” in the promoter regions of these genes (Figure 3A), suggesting a role for circadian pathways in SARS-CoV-2 RNA replication. To evaluate a role for BMAL1 in SARS-CoV-2 (Victoria 01/20 strain) replication, we infected *Bmal1* silenced Calu-3 and parental cells and observed a significant reduction in both intracellular and extracellular viral RNA along with infectious virus (Figure 3B). Importantly, we observed reduced replication of the alpha (B.1.1.7 strain) and beta (B.1.351 strain) variant of concern (VOC) in the *Bmal1* silenced cells (Figure 3C). We next assessed whether pharmacological inhibition of BMAL1 could restrict viral replication. Treating Calu-3 cells with SR9009 or the cryptochrome stabilizer KL001 which inhibits BMAL1 activity (Hirota et al., 2012) reduced SARS-CoV-2 RNA levels in a dose-dependent manner (Figure 3D), supporting a BMAL1-mediated effect. Given the link between circadian pathways and the cell cycle (Farshadi et al., 2020; Masri et al., 2013) that may influence SARS-CoV-2 infection (Bouhaddou et al., 2020; Su et al., 2020), we evaluated the effect of SR9009 or KL001 on the Calu-3 cell cycle and confirmed that neither drug had any detectable effect on cell cycle status under the experimental conditions used (Figure S4). Importantly, both drugs significantly reduced infection of ALI cultures of proximal airway epithelial cells (Figure 3E). Finally, we demonstrate that SR9009 treatment of Calu-3 cells inhibited the replication of both alpha B.1.1.7 and beta B.1.351 SARS-CoV-2 VOC (Figure 3F). These data show a role for BMAL1 in regulating SARS-CoV-2 RNA replication, including both alpha and beta VOC.

### BMAL1 regulates interferon-stimulated gene expression in lung epithelial cells

**Figure 4:**
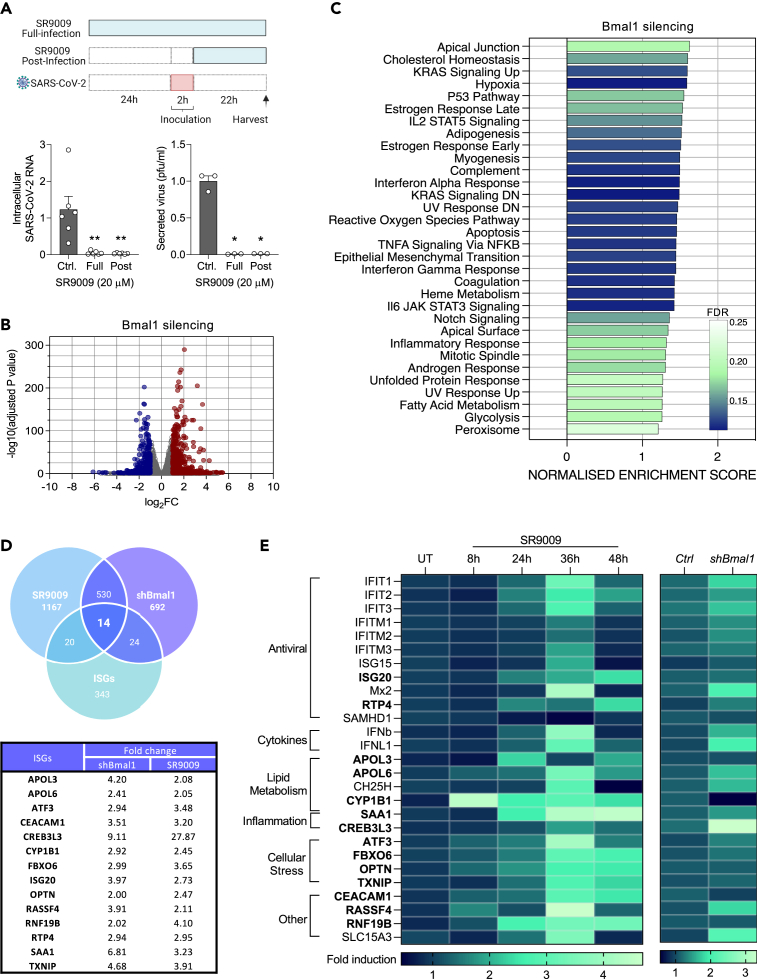
Transcriptomic analysis of *Bmal1* silenced and SR9009 treated cells show increased ISG response (A) Calu-3 cells were treated with SR9009 (20 μM) either 24h before infection or from 2h post-inoculation with SARS-CoV-2. Cells were incubated for 22h; intracellular viral RNA was quantified by qPCR and expressed relative to the untreated control. Infectivity of the extracellular virus was assessed by plaque assay using Vero-TMPRSS2 cells. Data are presented as mean ± S.E.M. from n = 3–6 independent biological replicates. Statistical significance was determined using Kruskal-Wallis ANOVA with Dunn's test. (B) Gene expression was quantified by RNA sequencing in *Bmal1* silenced or untreated Calu-3 cells. Differential expression analysis was performed between the conditions using DESeq2 Package. Volcano plot shows significantly differentially expressed genes based on a log_2_FC of ±1 and an adjusted (Benjamini Hochberg) p value of 0.05. Red points denote significant upregulation; blue denotes downregulation. (C) Gene set enrichment analysis (GSEA) investigated host pathways regulated by *Bmal1* silencing. Using the Hallmark gene sets from the molecular signatures database, 32 out of 50 gene sets were significantly upregulated in sh*Bmal1* above control cells, at an FDR of less than 25%. Significantly enriched hallmarks were plotted, ranked by normalized enrichment score, and colored by FDR. (D) Overlap of differentially expressed genes from *Bmal1* silenced and SR9009 treated Calu-3 cells with reported Interferon-stimulated genes (ISGs) (Kane et al., 2016). (E) ISG RNA levels of Calu-3 cells treated with SR9009 for 8, 24, 36 or 48h, and sh*Bmal1* Calu-3 cells were measured by qRT-PCR and normalized to TBP (TATA box binding protein, n = 3 biological replicates). The heatmaps display the fold induction relative to the untreated control. See related Figure S5. ∗p < 0.05; ∗∗p < 0.01.

To define whether circadian pathways regulate post-entry steps in the SARS-CoV-2 life cycle, we evaluated the effect of SR9009 on viral replication when added before or after viral inoculation. The agonist reduced intracellular RNA and secretion of infectious particles under both conditions (Figure 4A), leading us to conclude that both SARS-CoV-2 entry and replication are circadian regulated. To explore the mechanism underlying the antiviral phenotype, we sequenced RNA from *Bmal1*silenced and SR9009 treated Calu-3 cells. Differential expression analysis was performed, and Gene Set Enrichment Analysis (GSEA) (Subramanian et al., 2005) showed an enrichment in previously defined circadian gene sets from the molecular signatures database (MSigDB) (Liberzon et al., 2011) (Figures 4B, S5A, and S5B). GSEA investigated the host pathways regulated by BMAL1 or SR9009, and using the Hallmark gene sets from MSigDB (Liberzon et al., 2015), we found that 32 of 50 gene sets were enriched in the *Bmal1* silenced cells (Figure 4C) and 18 of 50 gene sets were altered by SR9009 treatment (Figure S4C). Genes involved in energy metabolism including fatty acid metabolism and cholesterol homeostasis, as well as hypoxia pathways, were enriched in both data sets. Interestingly, both cholesterol homeostasis and hypoxia pathways were previously linked to SARS-CoV-2 replication with pharmacological agents targeting these pathways showing antiviral activity (Saran et al., 2020; Wing et al., 2021; Wu et al., 2017).

Interferons (IFNs) constitute the first line of defense against viral infections by inducing the expression of various interferon-stimulated genes (ISGs). Emerging evidence highlights the importance of IFNs in controlling SARS-CoV-2 infection and disease outcomes (Arunachalam et al., 2020; Hadjadj et al., 2020; Zhang et al., 2020). We noted that IFN response pathways were significantly enriched in the *Bmal1* silenced Calu-3 cells (Figure 4C). A recent study reporting the circadian regulation of ISGs in murine skin (Greenberg et al., 2020) prompted us to investigate a link between BMAL1 and ISG expression in Calu-3 cells. We identified an overlap of 19 ISGs co-regulated by Bmal1 and SR9009 treatment with previously reported ISGs (Figure 4D) (Kane et al., 2016). To validate the RNA-seq data and to understand the kinetics of ISG induction, we treated Calu-3 cells with SR9009 for 8 h, 24 h, 36 h, and 48 h and quantified transcripts of selected ISGs, including those induced by SARS-CoV-2 infection or reported to show antiviral activity against SARS-CoV-2 (Martin-Sancho et al., 2021; Wang et al., 2020b). We noted a significant induction of most ISGs >36 h after treatment (Figure 4E), consistent with the antiviral activity of SR9009. In parallel, we showed ISG induction in *Bmal1* silenced Calu-3 cells, and this was not limited to ISGs with direct antiviral activity but includes those involved in many cellular pathways (Figure 4E).

## Discussion

Our studies show evidence for the key circadian component BMAL1 to regulate ACE2 in Calu-3 lung epithelial cells through post-transcriptional mechanisms, consistent with previous studies (Kojima et al., 2011; Reddy et al., 2006). There is an emerging role for microRNAs in modulating the circadian clock (Park et al., 2020; Pegoraro and Tauber, 2008), and miR-18 has been linked to ACE2 expression (Widiasta et al., 2020), providing a possible mechanism. Furthermore, we show striking inhibitory effects of *Bmal1* silencing, circadian modifying agents SR9009 and KL001 on SARS-CoV-2 entry, and virus replication. Of note, SARS-CoV-1 and alpha NL63 also require ACE2 to enter cells (Li, 2015), and our data support a role for the circadian regulation of these related coronaviruses. Circadian pathways are recognized to affect the pharmacokinetics and pharmacodynamics of drug responses (Ruben et al., 2019). Our observation that ACE2 is potentially a rhythmically “moving target” could be relevant to the evaluation of treatments targeting entry steps in SARS-CoV-2 life cycle.

In addition to the circadian regulation of ACE2-mediated viral entry, we observed a marked suppression of SARS-CoV-2 RNA and genesis of infectious particles following SR9009 and KL1001 treatment and in *Bmal1* silenced cells. Our bioinformatic analysis suggests that 30% of potential SARS-CoV-2 host factors are BMAL1/REV-ERB regulated, highlighting a role for circadian signaling to influence multiple steps in the viral life cycle. Further work is needed to characterize the circadian-dependent mechanisms of SARS-CoV-2 repression. However, our data suggest a role for BMAL1 to regulate ISGs which could impact SARS-CoV-2 replication. Namely, we can show that ISG20 and members of the Interferon Induced Protein With Tetratricopeptide Repeats 1 (IFIT) family that were previously reported to have antiviral activity against SARS-CoV-2 (Martin-Sancho et al., 2021) are induced in *Bmal1*silenced cells. In keeping with literature for other circadian-regulated inflammatory immune responses, this increase in ISG transcripts could be regulated at different stages of the IFN sensing and signaling pathways, via direct or indirect mechanisms. For example, some toll-like receptors (TLRs) are circadian regulated, with TLR9 having non-canonical E-box motifs (Silver et al., 2012). The interplay between IFNs and COVID-19 disease progression is complex, and despite initial findings, there is currently no clear evidence for a genetic immune predisposition to severe COVID-19 (Meisel et al., 2021; Povysil et al., 2021). Nevertheless, there is evidence at early stages of infection that SARS-CoV-2 is sensitive to ISG induction and IFNs are being discussed as a treatment option (Cheemarla et al., 2021; Hasselbalch et al., 2021; Yang et al., 2021). In this light, our findings could inform future therapeutic approaches.

A key finding from our study is the potential application of chronomodifying drugs for the treatment of SARS-CoV-2 infection (Sengupta et al., 2021). Dexamethasone can reduce the severity of COVID-19 (Group et al., 2021) and is known to synchronize circadian pathways (Oster et al., 2006; Pezuk et al., 2012). Over the last decade, a number of compounds that target core clock proteins have been developed (Ercolani et al., 2015), including REV-ERB (Everett and Lazar, 2014; Wang et al., 2020a) and ROR (Hirota et al., 2012; Solt and Burris, 2012) that we previously reported could inhibit hepatitis C virus and Human immunodeficiency virus 1 (HIV-1) replication (Borrmann et al., 2020; Zhuang et al., 2019). A report demonstrating REV-ERB-dependent and independent effects of SR9009 (Dierickx et al., 2019) suggests some additional off-target effects. We cannot exclude the possibility of additional pathways contributing to SR9009 anti-viral activity; however, our use of genetic targeting approaches confirms a role for BMAL1 in regulating SARS-CoV-2 replication. Since REV-ERBα agonists impact the host immune response by suppressing inflammatory mediators such as IL-6 (Gibbs et al., 2012), they offer a “two pronged” approach to reduce viral replication and adverse host immune responses.

Epidemiological (Pan et al., 2011; Vyas et al., 2012) and molecular evidence (Kervezee et al., 2018) shows that night shift workers suffer circadian disruption and are at an increased risk of developing chronic inflammatory diseases. A recent study reported that shift work was associated with a higher likelihood of in-hospital COVID-19 positivity (Maidstone et al., 2021). Our study raises questions as to whether the time of exposure to SARS-CoV-2 may impact on the likelihood of infection, the host response, virus shedding, transmission, and disease severity that are worthy of further investigation.

### Limitations of the study

A limitation of our study is our focus on the circadian regulation of SARS-CoV-2 replication in epithelial cells that lack the complex immune environment of the respiratory tract. Nevertheless, caveats remain to studying the circadian regulation of ACE2 in mice within the context of SARS-CoV-2. Firstly, murine ACE2 does not support SARS-CoV-2 entry; secondly, in the human ACE2 transgenic mouse, ACE2 transcription is not controlled by its endogenous promoter.

## STAR★Methods

### Key resources table

**Table:** 

REAGENT or RESOURCE	SOURCE	IDENTIFIER
**Antibodies**		

Rabbit anti-BMAL1	Abcam	Ab93806
Mouse anti-β-actin	Sigma	A5441
Rabbit anti-ACE2	Abcam	Cat#Ab108252; RRID:AB_10864415
Mouse anti-TMPRSS2	Santa Cruz Biotechnology	sc-515727
Anti-Spike FI-3A	Kind Gift from Prof Alain Townsend	FI-3A

**Bacterial and virus strains**		

SARS-CoV-2 Victoria 01/20, BVIC01 (Caly et al., 2020)	Public Health England	SARS-CoV-2 Victoria 01/20
SARS-CoV-2 B1.1.7: 20I/501Y.V1.HMPP1 (Tegally et al., 2020)	Public Health England	SARS-CoV-2 B1.1.7
SARS-CoV-2 B1.351: 201/501.V2.HV001 (Cele et al., 2021)	Centre for the AIDS Programme of Research in South Africa	SARS-CoV-2 B1.351

**Chemicals, peptides, and recombinant proteins**		

SR9009	Calbiochem	554726
KL001	Sigma	SML1032

**Critical commercial assays**		

CytoTox 96® Non-Radioactive Cytotoxicity Assay	Promega	G1780

**Experimental models: cell lines**		

Calu-3	Kind Gift from Professor Nicole Zitzmann	Calu-3
Vero E6	Kind Gift from Professor William James	Vero E6
Vero-TMPRSS2	Kind Gift from Dr. Makoto Takeda	Vero-TMPRSS2
Air Liquid Interface culture	Kind Gift from Dr. Tim Hinks	Air Liquid Interface culture

**Experimental models: organisms/strains**		

Mouse: Wild-type C57BL/6	Charles River	CR Strain code: 632

**Oligonucleotides**		

Primers	See Table S1	N/A

**Recombinant DNA**		

pSARS-Spike	Kind gift from Craig Thompson (University of Oxford)	N/A
p8.91 (GAG-POL)	Kind gift from Craig Thompson (University of Oxford)	N/A
pCSFW	Kind gift from Craig Thompson (University of Oxford)	N/A
pSpike-D614G	Kind gift from Ariel Isaacs and Naphak Modhiran (University of Queensland)	N/A
pSpike-Furin KO	Kind gift from Ariel Isaacs and Naphak Modhiran (University of Queensland)	N/A

**Software and algorithms**		

GraphPad 8	Prism	https://www.graphpad.com

### Resource availability

#### Lead contact

Further information and requests for resources and reagents should be directed to and will be fulfilled by the lead contact, Jane A McKeating (jane.mckeating@ndm.ox.ac.uk).

#### Materials availability

This study did not generate new unique reagents.

### Experimental model and subject details

#### Animals

Mouse experiments were carried out at the Institute of Viral and Liver Disease animal facility (approval number E-67-482-7). C57BL/6J male mice were purchased from Charles River and housed in individually ventilated cages under a 12/12 dark/light cycle with a ZT0 corresponding to 7am. After two weeks of acclimatisation, nine-week-old mice were sacrificed at different time points (ZT0, ZT4, ZT8, ZT12, ZT16, ZT20; n = 5/time point). Organs were harvested after exsanguination by intracardiac puncture, frozen in liquid nitrogen and kept at −80°C until further processing.

#### Cell culture

Calu-3, Huh-7, HEK293T and Vero E6 cells were cultured in DMEM supplemented with 10% fetal bovine serum (FBS), 2mM L-glutamine, 100 U/mL penicillin and 10μg/mL streptomycin (all reagents from Life Technologies/Thermo Fisher). Calu-3 was a kind gift from Dr Anderson Ryan (Oncology Department, University of Oxford). Vero-TMPRSS2 cells (a Vero E6 cell line stably overexpressing the TMPRSS2 gene, kindly provided by Dr Makoto Takeda at Department of Virology III, National Institute of Infectious Diseases (Matsuyama et al., 2020)) were cultured in DMEM supplemented with 10% fetal bovine serum, 10 units/mL penicillin, 10 mg/mL streptomycin, 10 mM HEPES (pH 7.4), and 1 mg/mL G418 (Life Technologies, UK). All cell lines were maintained at 37°C and 5% CO_2_ in a standard culture incubator. For Air Liquid Interface (ALI) culture, primary bronchial epithelial cells were seeded onto 0.4 μm transwells (Greiner Bio-One, Frickenhausen, Germany) and cultured in Promocell Airway Epithelial Cell Growth media until confluent. Media was removed and cells fed basally with Pneumacult-ALI media (StemCell Technologies, Vancouver, Canada) for 3 months to ensure complete differentiation to ALI with uniform ciliary beating and mucus production visible. Human primary bronchial epithelial cells were obtained using flexible fibreoptic bronchoscopy from healthy control volunteers under light sedation with fentanyl and midazolam. Participants provided written informed consent. The study was reviewed by the Oxford Research Ethics Committee B (18/SC/0361).

#### SARS-CoV-2 strains

Victoria 01/20 (BVIC01) (Caly et al., 2020) (provided by PHE Porton Down after supply from the Doherty Centre Melbourne, Australia); B.1.1.7 (Tegally et al., 2020) (20I/501Y.V1.HMPP1) (provided by PHE Porton Down) and B.1.351 (201/501.V2.HV001) (Cele et al., 2021) (Centre for the AIDS Programme of Research in South Africa) were passaged in Vero E6 cells.

### Method details

#### Materials

All reagents and chemicals were obtained from Sigma-Aldrich (now Merck) unless stated otherwise. REV-ERB agonist SR9009 was purchased from Calbiochem, US, dissolved in dimethyl sulfoxide (DMSO) and cytotoxicity determined by a Lactate dehydrogenase (LDH) assay (Promega, UK) or MTT assay (Sigma, UK). The lenti-shBmal1 plasmid was purchased from Abmgood (UK). Recombinant ACE2-Fc was previously reported (Zhou et al., 2020). The BMAL1 promoter luciferase reporter vector was purchased from Addgene, UK (Plasmid #46824) and contains 1 kb of BMAL1 upstream region and 53 nucleotides of exon 1, fused in-frame to the luciferase coding region followed by 1 kb of Bmal1 3′UTR. This construct has been widely used in circadian research (Brown et al., 2005; Bu et al., 2018; Sanghani et al., 2020; Schmitt et al., 2018). The Cry1 promoter construct and BMAL1/CLOCK expression plasmids were a gift of Ximing Qin, Anhui University, Hefei, China. The Cry1 promoter was amplified from genomic DNA using forward primer: 5′-ATCCTCGAGGTAAAGATGCACATGTGGCCCTG-3′ and reverse primer: 5′-CTAAAGCTTCGTCCGGAGGACACGCATACC-3′ and cloned into the pGL3 luciferase reporter vector (Promega, UK). The Bmal1 expression plasmid was previously described (Yang et al., 2020) and further engineered with a Flag tag. The Clock expression plasmid was previously described (Schoenhard et al., 2003).

#### SARS-CoV-2 pseudoparticle genesis and infection

SARS-CoV-2 lentiviral pp were generated by transfecting HEK-293T cells with p8.91 (Gag-pol), pCSFW (luciferase reporter) and a codon optimized expression construct pcDNA3.1-SARS-CoV-2-Spike, as previously reported (Thompson et al., 2020). The Furin cleavage site mutant was generated by mutagenesis of a pcDNA3.1 based clone expressing a C-terminally flag-tagged SARS-CoV-2 Spike protein (Wuhan-Hu-1 isolate; MN908947.3). The polybasic cleavage site TNSPRRA in SARS-CoV-2 Spike was replaced with the corresponding SARS-CoV variant sequence SLL. The pNBF SARS-CoV2 FL D614G mutant was a kind gift from Dr. Daniel Watterson and Dr. Naphak Modhiran at the University of Queensland. Supernatants containing viral pp were harvested at 48 and 72h post-transfection, frozen and stored at −80°C. As a control pp were generated that lacked a viral envelope glycoprotein and were included in all infection experiments to control for non-specific uptake. This control was included in all pp experiments and the luciferase values subtracted from values acquired with the SARS-CoV-2pp. To confirm spike-dependent infection, SARS-CoV-2pp were incubated with the anti-S-mAb FI-3A (1 μg/mL) (Huang et al., 2020) for 30 min prior to infection for all experiments.

SARS-CoV-2 VSV pp were generated as previously reported (Hoffmann et al., 2020b) using reagents provided by Stefan Pöhlmann (Infection biology unit, German Primate Center, Göttingen, Germany). Briefly, HEK-293T cells were transfected with an expression construct encoding a c-terminal truncated version of SARS-CoV-2-S for assembly into VSV pp (pCG1-SARS-CoV-2-S-ΔC) (Hoffmann et al., 2020a). After 24h, the transfected cells were infected with a replication-incompetent VSV (VSV∗ΔG) (Berger Rentsch and Zimmer, 2011) containing GFP and firefly luciferase reporter genes, provided by Gert Zimmer (Institute of Virology and Immunology, Mittelhäusern, Switzerland). After 1h, the cells were washed with PBS before medium containing a VSV-G antibody (I1, mouse hybridoma supernatant from CRL-2700, ATCC) and supernatants harvested after 24h. The VSV∗ΔG used for generating the pps was propagated in BHK-21 G43 cells stably expressing VSV-G. Viral titers were determined by infecting Calu-3 cells and measuring cellular luciferase after 48h.

#### SARS-CoV-2 propagation and infection

For infection experiments SARS-CoV-2 (Victoria 01/20 isolate) was propagated in Vero E6 cells. Naïve Vero E6 cells were infected with SARS-CoV-2 at an MOI of 0.003 and incubated for 48-72h until visible cytopathic effects were observed. Culture supernatants were harvested, clarified by centrifugation to remove residual cell debris and stored at −80°C before measuring the infectious titer by plaque assay. Briefly, Vero-TMPRSS2 cells were inoculated with serial dilutions of SARS-CoV-2 stocks for 2h followed by addition of a semi-solid overlay consisting of 3% carboxymethyl cellulose (SIGMA). Cells were incubated for 72h, plaques enumerated by fixing cells using amido black stain and plaque-forming units (PFU) per mL calculated. For infection of Calu-3 cells, cells were plated 24h before infecting with the stated MOI. Cells were inoculated with virus for 2h after which the unbound virus was removed by washing three times with phosphate buffered saline (PBS). ALI primary bronchial epithelial cultures were inoculated with SARS-CoV-2 via the apical surface for 2h and unbound virus removed by washing three times with pre-warmed PBS. Unless otherwise stated, infected cells were maintained for 24h before harvesting for downstream applications.

#### SARS-CoV-2 cell-cell fusion assay

The SARS-CoV-2 cell-cell fusion assay was performed as previously described (Thakur et al., 2021). Briefly, HEK-293T Lenti rLuc-GFP 1–7 (effector cells) and Huh-7.5 Lenti rLuc-GFP 8–11 (target cells) cells were seeded separately at 7.5×10^5^ per well in a 6 well dish in 3mL of phenol red free DMEM, supplemented with 10% FBS, 1% sodium pyruvate and 1% penicillin/streptomycin (10,000 U/mL) before culturing overnight at 37°C, 5% CO_2_. The effector cells were transfected with a plasmid expressing SARS-CoV-2 Spike or a blank vector. Meanwhile, target cells were diluted to 2×10^5^/mL and 100μL seeded into a clear, flat-bottomed 96 well plate and mock-treated or treated with SR9009 (3μM or 7μM). The following day, the effector cells were harvested, diluted to 2×10^5^/mL and 100μL cultured with the drug/target cell mix, with SR9009 concentrations maintained at 3μM or 7μM. To quantify GFP expression, cells were imaged every 4h using an IncuCyte S3 live cell imaging system (Essen BioScience). Five fields of view were obtained per well at 10× magnification and GFP expression determined by calculating the total GFP area using the IncuCyte analysis software.

#### Cell cycle analysis

Calu-3 cells were incubated in 10 μM bromodeoxyuridine (BrdU) (Sigma) for 60 min at 37°C. Cells were then washed, and trypsinised before fixation in ice-cold ethanol. Cells underwent 30 min digestion in warmed pepsin solution (Sigma), before treating with 2M HCl for 15 min. Samples were washed, then blocked (0.5% BSA/0.5% Tween 20) for 30 min before staining with a-BrdU-488 (Biolegend) directly conjugated antibody and Propidium Iodide (Invitrogen). Samples were acquired on BD Cyan Flow Cytometer and analyzed via Flow-Jo.

#### Oligonucleotides

Provided in Table S1.

##### RT-qPCR

Cells were washed in PBS then lysed using Tri-reagent (Sigma), and mRNA extracted by phase separation or RNeasy kit (Qiagen). Equal amounts of cDNA were synthesized using the High Capacity cDNA Kit (Applied Biosystems) and mRNA expression determined using Fast SYBR master mix in a StepOne thermocycler (Applied Biosystems) using the ΔΔCt method. The lung tissues were lysed in TRI Reagent (Sigma) using a gentleMACS dissociator and RNA extracted using the manufacturers protocol. cDNAs were synthesized using the Maxima H Minus cDNA Synthesis Kit (Thermo Scientific) and mRNA quantified using iTaq Universal SYBR Green Supermix (Biorad) and specific primers on a QuantStudio 3 RT-PCR system (Applied Biosystems).

##### Immunoblotting

Cell lysates were prepared by washing cells with PBS, then lysed in Igepal lysis buffer (10mM Tris pH 7.5, 0.25M NaCl, 0.5% Igepal) supplemented with protease inhibitor cocktail (Roche Complete™) at 4°C for 5 min, followed by clarification by centrifugation (3 min, 12,000 rpm). Supernatant was mixed with Laemmli sample buffer, separated by SDS-PAGE and proteins transferred to polyvinylidene difluoride membrane (Immobilon-P, Millipore). Membranes were blocked in 5% milk in PBS/0.1% Tween 20, then incubated with anti-ACE2 (Abcam ab108252), anti-TMPRSS2 (SCBT sc-515727), anti-BMAL1 (Abcam Ab93806) or anti-β-actin (Sigma A5441) primary antibodies and appropriate HRP-conjugated secondary antibodies (DAKO). Chemiluminescence substrate (West Dura, 34076, Thermo Fisher Scientific) was used to visualize proteins using a ChemiDoc XRS + imaging system (BioRad). Anti-β-actin-HRP conjugate (Abcam ab49900) and/or Coomassie brilliant blue staining was used to verify equal protein loading and densitometric analysis performed using ImageJ software (NIH).

##### Bioinformatics

The published 153 SARS-CoV-2 host factors were converted to Entrez gene names (Daniloski et al., 2021). BMAL1 regulated genes were obtained from the published BMAL1 ChIP-seq in the mouse liver (Beytebiere et al., 2019). REV-ERB regulated genes were defined from published liver-specific loss of the REV-ERB paralogues (Cho et al., 2012). Promoters (-1kb from TSS) of genes encoding SARS-CoV-2 host factors were analyzed with the HOMER (Hypergeometric Optimization of Motif EnRichment) tool for motif discovery (E-box motif CANNTG; RORE motif RGGTCA). Gene set enrichment analysis was performed using GSEA_4.1.0, and gene sets of interest retrieved from the Molecular Signatures Database. Analysis was carried out using Phenotype grouping between shBmal1 and untreated Calu-3 cells, with 1000 permutations.

##### RNA-seq analysis

*Bmal1* silenced, SR9009 treated and control Calu-3 cells were harvested for RNA isolation and RNA-sequencing at Novogene. RNA purity was assessed with a NanoDrop 2000 spectrophotometer (Thermo Fisher Scientific) and integrity determined using a 2100 Bioanalyzer Instrument (Agilent Technologies). Sequence adapters were removed and reads trimmed by Trim Galore v0.5.079. The reads were mapped against the reference reference human genome (hg38/GRCm38) using STAR v2.5.380. Counts per gene were calculated using Rsubread v1.28.181. Reads were analyzed by edgeR v3.30.082, normalized using TMM, counts per million calculated and differential expression analysis performed.

##### Chromatin immuno-precipitation (ChIP) and quantitative PCR

1×10^7^ Calu-3 cells were harvested from 80% confluent 15cm plates and fixed with 1% formaldehyde (Sigma Aldrich 47608) for 10 min at room temperature before quenching with 125 mM glycine. Cells were washed twice with ice cold PBS, pelleted (800rpm, 10 min 4°C) and lysed in 500μl of Nuclear Extraction buffer (10 mM Tris pH 8.0, 10 mM NaCl, 1% NP-40) supplemented with a protease inhibitor cocktail (Roche Complete™). Samples were diluted 1:1 in ChIP Dilution Buffer (0.01% SDS, 1.1% Triton, 0.2mM EDTA; 16.7 mM Tris pH8.1, 167mM NaCl) and pulse sonicated using a Bioruptor R sonicator (Diagenode, U.K.) at high power for 15 min at 4°C (15 s on, 15 s off). Sonicated lysates were clarified by centrifugation at 1300 rpm for 10 min and precleared with Protein A agarose beads (Millipore, 16–156). Samples were immunoprecipitated with primary anti-BMAL1 (ChIP grade, Abcam Ab3350) or anti-REV-ERBα (ChIP grade, Abcam Ab181604) or IgG control mAb and precipitated with Protein A agarose beads. Precipitates were washed in low salt buffer (0.1% SDS, 1% Triton, 2 mM EDTA, 20 mM Tris pH8.1, 150mM NaCl), high salt buffer (0.1% SDS, 1% Triton, 2mM EDTA, 20 mM Tris pH 8.1, 500 mM NaCl), LiCl Buffer (1% Igepal, 1mM EDTA, 10 mM Tris pH 8.1, 250 mM LiCl, 1% sodium deoxycholate) and finally twice in TE wash buffer (10 mM Tris pH8.0, 1mM EDTA) before being eluted from the beads in 240μL of elution buffer (0.1 M NaHCO_3_, 1% SDS). Complexes were reverse crosslinked in a heated shaker at 65°C overnight, 1400 rpm, in the presence of 200 mM NaCl. Eluates were treated with Proteinase K (SIGMA) and RNaseA (SIGMA) before cleanup using MiniElute PCR Purification columns (Qiagen). Samples were analyzed on a LightCycler96 (Roche) using SYBR green qPCR mastermix (PCR Biosystems, UK). Fold enrichment was calculated for each sample relative to their own IgG controls.

### Quantification and statistical analysis

Data was analyzed using GraphPad Prism version 8.0.2 (GraphPad, San Diego, CA, (USA). p values <0.05 were considered significant; significance values are indicated as ∗p < 0.05; ∗∗p < 0.01; ∗∗∗p < 0.001; ∗∗∗∗p < 0.0001. Please see individual figure legends for further details.

## Data Availability

The authors declare that all data supporting the findings of this study are available in the article along with supplementary Information file and source data. The RNA-seq data from sh*Bmal1* silenced or SR9009 treated Calu-3 cells are deposited at NCBI (Database number: GSE176393).

## References

[bib1] Arunachalam P.S., Wimmers F., Mok C.K.P., Perera R., Scott M., Hagan T., Sigal N., Feng Y., Bristow L., Tak-Yin Tsang O. (2020). Systems biological assessment of immunity to mild versus severe COVID-19 infection in humans. Science.

[bib2] Berger Rentsch M., Zimmer G. (2011). A vesicular stomatitis virus replicon-based bioassay for the rapid and sensitive determination of multi-species type I interferon. PLoS ONE.

[bib3] Beytebiere J.R., Trott A.J., Greenwell B.J., Osborne C.A., Vitet H., Spence J., Yoo S.H., Chen Z., Takahashi J.S., Ghaffari N., Menet J.S. (2019). Tissue-specific BMAL1 cistromes reveal that rhythmic transcription is associated with rhythmic enhancer-enhancer interactions. Genes Dev..

[bib4] Borrmann H., Davies R., Dickinson M., Pedroza-Pacheco I., Schilling M., Vaughan-Jackson A., Magri A., James W., Balfe P., Borrow P. (2020). Pharmacological activation of the circadian component REV-ERB inhibits HIV-1 replication. Sci. Rep..

[bib5] Bouhaddou M., Memon D., Meyer B., White K.M., Rezelj V.V., Correa Marrero M., Polacco B.J., Melnyk J.E., Ulferts S., Kaake R.M. (2020). The global phosphorylation landscape of SARS-CoV-2 infection. Cell.

[bib6] Brown S.A., Fleury-Olela F., Nagoshi E., Hauser C., Juge C., Meier C.A., Chicheportiche R., Dayer J.M., Albrecht U., Schibler U. (2005). The period length of fibroblast circadian gene expression varies widely among human individuals. Plos Biol..

[bib7] Bu Y., Yoshida A., Chitnis N., Altman B.J., Tameire F., Oran A., Gennaro V., Armeson K.E., McMahon S.B., Wertheim G.B. (2018). A PERK-miR-211 axis suppresses circadian regulators and protein synthesis to promote cancer cell survival. Nat. Cell Biol..

[bib8] Caly L., Druce J., Roberts J., Bond K., Tran T., Kostecki R., Yoga Y., Naughton W., Taiaroa G., Seemann T. (2020). Isolation and rapid sharing of the 2019 novel coronavirus (SARS-CoV-2) from the first patient diagnosed with COVID-19 in Australia. Med. J. Aust..

[bib9] Cao Y., Xu X., Kitanovski S., Song L., Wang J., Hao P., Hoffmann D. (2021). Comprehensive comparison of RNA-seq data of SARS-CoV-2, SARS-CoV and MERS-CoV infections: alternative entry routes and innate immune responses. Front Immunol..

[bib10] Cele S., Gazy I., Jackson L., Hwa S.-H., Tegally H., Lustig G., Giandhari J., Pillay S., Wilkinson E., Naidoo Y. (2021). Escape of SARS-CoV-2 501Y.V2 variants from neutralization by convalescent plasma. medRxiv.

[bib11] Cheemarla N.R., Watkins T.A., Mihaylova V.T., Wang B., Zhao D., Wang G., Landry M.L., Foxman E.F. (2021). Magnitude and timing of the antiviral response determine SARS-CoV-2 replication early in infection. medRxiv.

[bib12] Cho H., Zhao X., Hatori M., Yu R.T., Barish G.D., Lam M.T., Chong L.W., DiTacchio L., Atkins A.R., Glass C.K. (2012). Regulation of circadian behaviour and metabolism by REV-ERB-alpha and REV-ERB-beta. Nature.

[bib13] Chu H., Chan J.F., Yuen T.T., Shuai H., Yuan S., Wang Y., Hu B., Yip C.C., Tsang J.O., Huang X. (2020). Comparative tropism, replication kinetics, and cell damage profiling of SARS-CoV-2 and SARS-CoV with implications for clinical manifestations, transmissibility, and laboratory studies of COVID-19: an observational study. Lancet Microbe.

[bib14] Daniloski Z., Jordan T.X., Wessels H.H., Hoagland D.A., Kasela S., Legut M., Maniatis S., Mimitou E.P., Lu L., Geller E. (2021). Identification of required host factors for SARS-CoV-2 infection in human cells. Cell.

[bib15] Davies N.G., Abbott S., Barnard R.C., Jarvis C.I., Kucharski A.J., Munday J.D., Pearson C.A.B., Russell T.W., Tully D.C., Washburne A.D. (2021). Estimated transmissibility and impact of SARS-CoV-2 lineage B.1.1.7 in England. Science.

[bib16] Dierickx P., Emmett M.J., Jiang C., Uehara K., Liu M., Adlanmerini M., Lazar M.A. (2019). SR9009 has REV-ERB-independent effects on cell proliferation and metabolism. Proc. Natl. Acad. Sci. U S A.

[bib17] Early J.O., Menon D., Wyse C.A., Cervantes-Silva M.P., Zaslona Z., Carroll R.G., Palsson-McDermott E.M., Angiari S., Ryan D.G., Corcoran S.E. (2018). Circadian clock protein BMAL1 regulates IL-1beta in macrophages via NRF2. Proc. Natl. Acad. Sci. U S A.

[bib18] Edgar R.S., Stangherlin A., Nagy A.D., Nicoll M.P., Efstathiou S., O'Neill J.S., Reddy A.B. (2016). Cell autonomous regulation of herpes and influenza virus infection by the circadian clock. Proc. Natl. Acad. Sci. U S A.

[bib19] Ehlers A., Xie W., Agapov E., Brown S., Steinberg D., Tidwell R., Sajol G., Schutz R., Weaver R., Yu H. (2018). BMAL1 links the circadian clock to viral airway pathology and asthma phenotypes. Mucosal Immunol..

[bib20] Ercolani L., Ferrari A., De Mei C., Parodi C., Wade M., Grimaldi B. (2015). Circadian clock: time for novel anticancer strategies?. Pharmacol. Res..

[bib21] Everett L.J., Lazar M.A. (2014). Nuclear receptor Rev-erbalpha: up, down, and all around. Trends Endocrinol. Metabol. TEM.

[bib22] Farshadi E., van der Horst G.T.J., Chaves I. (2020). Molecular links between the circadian clock and the cell cycle. J. Mol. Biol..

[bib23] Gavriatopoulou M., Korompoki E., Fotiou D., Ntanasis-Stathopoulos I., Psaltopoulou T., Kastritis E., Terpos E., Dimopoulos M.A. (2020). Organ-specific manifestations of COVID-19 infection. Clin. Exp. Med..

[bib24] Gibbs J., Ince L., Matthews L., Mei J., Bell T., Yang N., Saer B., Begley N., Poolman T., Pariollaud M. (2014). An epithelial circadian clock controls pulmonary inflammation and glucocorticoid action. Nat. Med..

[bib25] Gibbs J.E., Blaikley J., Beesley S., Matthews L., Simpson K.D., Boyce S.H., Farrow S.N., Else K.J., Singh D., Ray D.W., Loudon A.S. (2012). The nuclear receptor REV-ERBalpha mediates circadian regulation of innate immunity through selective regulation of inflammatory cytokines. Proc. Natl. Acad. Sci. U S A.

[bib26] Gillim-Ross L., Taylor J., Scholl D.R., Ridenour J., Masters P.S., Wentworth D.E. (2004). Discovery of novel human and animal cells infected by the severe acute respiratory syndrome coronavirus by replication-specific multiplex reverse transcription-PCR. J. Clin. Microbiol..

[bib27] Greenberg E.N., Marshall M.E., Jin S., Venkatesh S., Dragan M., Tsoi L.C., Gudjonsson J.E., Nie Q., Takahashi J.S., Andersen B. (2020). Circadian control of interferon-sensitive gene expression in murine skin. Proc. Natl. Acad. Sci. U S A.

[bib28] Group R.C., Horby P., Lim W.S., Emberson J.R., Mafham M., Bell J.L., Linsell L., Staplin N., Brightling C., Ustianowski A. (2021). Dexamethasone in hospitalized patients with Covid-19. N. Engl. J. Med..

[bib29] Guan W.J., Liang W.H., Zhao Y., Liang H.R., Chen Z.S., Li Y.M., Liu X.Q., Chen R.C., Tang C.L., Wang T. (2020). Comorbidity and its impact on 1590 patients with COVID-19 in China: a nationwide analysis. Eur. Respir. J..

[bib30] Hadjadj J., Yatim N., Barnabei L., Corneau A., Boussier J., Smith N., Pere H., Charbit B., Bondet V., Chenevier-Gobeaux C. (2020). Impaired type I interferon activity and inflammatory responses in severe COVID-19 patients. Science.

[bib31] Hamming I., Timens W., Bulthuis M.L., Lely A.T., Navis G., van Goor H. (2004). Tissue distribution of ACE2 protein, the functional receptor for SARS coronavirus. A first step in understanding SARS pathogenesis. J. Pathol..

[bib32] Harding H.P., Lazar M.A. (1993). The orphan receptor Rev-ErbA alpha activates transcription via a novel response element. Mol. Cell. Biol..

[bib33] Hasselbalch H.C., Skov V., Kjaer L., Ellervik C., Poulsen A., Poulsen T.D., Nielsen C.H. (2021). COVID-19 as a mediator of interferon deficiency and hyperinflammation: rationale for the use of JAK1/2 inhibitors in combination with interferon. Cytokine Growth Factor Rev..

[bib34] Hirota T., Lee J.W., St John P.C., Sawa M., Iwaisako K., Noguchi T., Pongsawakul P.Y., Sonntag T., Welsh D.K., Brenner D.A. (2012). Identification of small molecule activators of cryptochrome. Science.

[bib35] Hoffmann M., Kleine-Weber H., Pohlmann S. (2020). A multibasic cleavage site in the spike protein of SARS-CoV-2 is essential for infection of human lung cells. Mol. Cell.

[bib36] Hoffmann M., Kleine-Weber H., Schroeder S., Kruger N., Herrler T., Erichsen S., Schiergens T.S., Herrler G., Wu N.H., Nitsche A. (2020). SARS-CoV-2 cell entry depends on ACE2 and TMPRSS2 and is blocked by a clinically proven protease inhibitor. Cell.

[bib37] Hou Y.J., Okuda K., Edwards C.E., Martinez D.R., Asakura T., Dinnon K.H., Kato T., Lee R.E., Yount B.L., Mascenik T.M. (2020). SARS-CoV-2 reverse genetics reveals a variable infection gradient in the respiratory tract. Cell.

[bib38] Huang K.-Y.A., Tan T.K., Chen T.-H., Huang C.-G., Harvey R., Hussain S., Chen C.-P., Harding A., Gilbert-Jaramillo J., Liu X. (2020). Plasmablast-derived antibody response to acute SARS-CoV-2 infection in humans. BioRxiv.

[bib39] Ince L.M., Zhang Z., Beesley S., Vonslow R.M., Saer B.R., Matthews L.C., Begley N., Gibbs J.E., Ray D.W., Loudon A.S.I. (2019). Circadian variation in pulmonary inflammatory responses is independent of rhythmic glucocorticoid signaling in airway epithelial cells. FASEB J. Off. Publ. Fed. Am. Societies Exp. Biol..

[bib40] Johnson B.A., Xie X., Bailey A.L., Kalveram B., Lokugamage K.G., Muruato A., Zou J., Zhang X., Juelich T., Smith J.K. (2021). Loss of furin cleavage site attenuates SARS-CoV-2 pathogenesis. Nature.

[bib41] Kane M., Zang T.M., Rihn S.J., Zhang F., Kueck T., Alim M., Schoggins J., Rice C.M., Wilson S.J., Bieniasz P.D. (2016). Identification of interferon-stimulated genes with antiretroviral activity. Cell Host Microbe.

[bib42] Kervezee L., Cuesta M., Cermakian N., Boivin D.B. (2018). Simulated night shift work induces circadian misalignment of the human peripheral blood mononuclear cell transcriptome. Proc. Natl. Acad. Sci. U S A.

[bib43] Kidd M., Richter A., Best A., Cumley N., Mirza J., Percival B., Mayhew M., Megram O., Ashford F., White T. (2021). S-variant SARS-CoV-2 lineage B1.1.7 is associated with significantly higher viral load in samples tested by TaqPath polymerase chain reaction. J. Infect Dis..

[bib44] Kissler S.M., Fauver J.R., Mack C., Tai C.G., Breban M.I., Watkins A.E., Samant R.M., Anderson D.J., Ho D.D., Metti J. (2021). Densely sampled viral trajectories suggest longer duration of acute infection with B.1.1.7 variant relative to non-B.1.1.7 SARS-CoV-2. medRxiv.

[bib45] Kojima S., Shingle D.L., Green C.B. (2011). Post-transcriptional control of circadian rhythms. J. Cell Sci.

[bib46] Korber B., Fischer W.M., Gnanakaran S., Yoon H., Theiler J., Abfalterer W., Hengartner N., Giorgi E.E., Bhattacharya T., Foley B. (2020). Tracking changes in SARS-CoV-2 spike: evidence that D614G increases infectivity of the COVID-19 virus. Cell.

[bib47] Lambert D.W., Yarski M., Warner F.J., Thornhill P., Parkin E.T., Smith A.I., Hooper N.M., Turner A.J. (2005). Tumor necrosis factor-alpha convertase (ADAM17) mediates regulated ectodomain shedding of the severe-acute respiratory syndrome-coronavirus (SARS-CoV) receptor, angiotensin-converting enzyme-2 (ACE2). J. Biol. Chem..

[bib48] Li F. (2015). Receptor recognition mechanisms of coronaviruses: a decade of structural studies. J. Virol..

[bib49] Li Y., Renner D.M., Comar C.E., Whelan J.N., Reyes H.M., Cardenas-Diaz F.L., Truitt R., Tan L.H., Dong B., Alysandratos K.D. (2021). SARS-CoV-2 induces double-stranded RNA-mediated innate immune responses in respiratory epithelial-derived cells and cardiomyocytes. Proc. Natl. Acad. Sci. U S A.

[bib50] Liberzon A., Birger C., Thorvaldsdottir H., Ghandi M., Mesirov J.P., Tamayo P. (2015). The Molecular Signatures Database (MSigDB) hallmark gene set collection. Cell Syst.

[bib51] Liberzon A., Subramanian A., Pinchback R., Thorvaldsdottir H., Tamayo P., Mesirov J.P. (2011). Molecular signatures database (MSigDB) 3.0. Bioinformatics.

[bib52] Maidstone R., Anderson S.G., Ray D.W., Rutter M.K., Durrington H.J., Blaikley J.F. (2021). Shift work is associated with positive COVID-19 status in hospitalised patients. Thorax.

[bib53] Maiese K. (2020). Circadian clock genes: targeting innate immunity for antiviral strategies against COVID-19. Curr. Neurovasc Res..

[bib54] Marshall M. (2020). How COVID-19 can damage the brain. Nature.

[bib55] Martin-Sancho L., Lewinski M.K., Pache L., Stoneham C.A., Yin X., Becker M.E., Pratt D., Churas C., Rosenthal S.B., Liu S. (2021). Functional landscape of SARS-CoV-2 cellular restriction. Mol. Cell.

[bib56] Masri S., Cervantes M., Sassone-Corsi P. (2013). The circadian clock and cell cycle: interconnected biological circuits. Curr. Opin. Cell Biol..

[bib57] Matsuyama S., Nao N., Shirato K., Kawase M., Saito S., Takayama I., Nagata N., Sekizuka T., Katoh H., Kato F. (2020). Enhanced isolation of SARS-CoV-2 by TMPRSS2-expressing cells. Proc. Natl. Acad. Sci. U S A.

[bib58] Meisel C., Akbil B., Meyer T., Lankes E., Corman V.M., Staudacher O., Unterwalder N., Kolsch U., Drosten C., Mall M.A. (2021). Mild COVID-19 despite autoantibodies against type I IFNs in autoimmune polyendocrine syndrome type 1. J. Clin. Invest..

[bib59] Mure L.S., Le H.D., Benegiamo G., Chang M.W., Rios L., Jillani N., Ngotho M., Kariuki T., Dkhissi-Benyahya O., Cooper H.M., Panda S. (2018). Diurnal transcriptome atlas of a primate across major neural and peripheral tissues. Science.

[bib60] Nie Y., Wang P., Shi X., Wang G., Chen J., Zheng A., Wang W., Wang Z., Qu X., Luo M. (2004). Highly infectious SARS-CoV pseudotyped virus reveals the cell tropism and its correlation with receptor expression. Biochem. Biophys. Res. Commun..

[bib61] Oster H., Damerow S., Kiessling S., Jakubcakova V., Abraham D., Tian J., Hoffmann M.W., Eichele G. (2006). The circadian rhythm of glucocorticoids is regulated by a gating mechanism residing in the adrenal cortical clock. Cell Metab.

[bib62] Pan A., Schernhammer E.S., Sun Q., Hu F.B. (2011). Rotating night shift work and risk of type 2 diabetes: two prospective cohort studies in women. Plos Med..

[bib63] Pariollaud M., Gibbs J.E., Hopwood T.W., Brown S., Begley N., Vonslow R., Poolman T., Guo B., Saer B., Jones D.H. (2018). Circadian clock component REV-ERBalpha controls homeostatic regulation of pulmonary inflammation. J. Clin. Invest..

[bib64] Park I., Kim D., Kim J., Jang S., Choi M., Choe H.K., Choe Y., Kim K. (2020). microRNA-25 as a novel modulator of circadian Period2 gene oscillation. Exp. Mol. Med..

[bib65] Pegoraro M., Tauber E. (2008). The role of microRNAs (miRNA) in circadian rhythmicity. J. Genet..

[bib66] Pezuk P., Mohawk J.A., Wang L.A., Menaker M. (2012). Glucocorticoids as entraining signals for peripheral circadian oscillators. Endocrinology.

[bib67] Povysil G., Butler-Laporte G., Shang N., Wang C., Khan A., Alaamery M., Nakanishi T., Zhou S., Forgetta V., Eveleigh R.J. (2021). Rare loss-of-function variants in type I IFN immunity genes are not associated with severe COVID-19. J. Clin. Invest..

[bib68] Ray S., Reddy A.B. (2020). COVID-19 management in light of the circadian clock. Nat. Rev. Mol. Cell Biol..

[bib69] Reddy A.B., Karp N.A., Maywood E.S., Sage E.A., Deery M., O'Neill J.S., Wong G.K., Chesham J., Odell M., Lilley K.S. (2006). Circadian orchestration of the hepatic proteome. Curr. Biol..

[bib70] Rees-Spear C., Muir L., Griffith S.A., Heaney J., Aldon Y., Snitselaar J.L., Thomas P., Graham C., Seow J., Lee N. (2021). The effect of spike mutations on SARS-CoV-2 neutralization. Cell Rep..

[bib71] Ruben M.D., Smith D.F., FitzGerald G.A., Hogenesch J.B. (2019). Dosing time matters. Science.

[bib72] Sanders D.W., Jumper C.C., Ackerman P.J., Bracha D., Donlic A., Kim H., Kenney D., Castello-Serrano I., Suzuki S., Tamura T. (2021). SARS-CoV-2 requires cholesterol for viral entry and pathological syncytia formation. Elife.

[bib73] Sanghani H.R., Jagannath A., Humberstone T., Ebrahimjee F., Thomas J.M., Churchill G.C., Cipriani A., Attenburrow M.J., Perestenko O.V., Cowley S.A. (2020). Patient fibroblast circadian rhythms predict lithium sensitivity in bipolar disorder. Mol. Psychiatry.

[bib74] Saran A.R., Dave S., Zarrinpar A. (2020). Circadian rhythms in the pathogenesis and treatment of fatty liver disease. Gastroenterology.

[bib75] Scheiermann C., Gibbs J., Ince L., Loudon A. (2018). Clocking in to immunity. Nat. Rev. Immunol..

[bib76] Scheiermann C., Kunisaki Y., Lucas D., Chow A., Jang J.E., Zhang D., Hashimoto D., Merad M., Frenette P.S. (2012). Adrenergic nerves govern circadian leukocyte recruitment to tissues. Immunity.

[bib77] Schmitt K., Grimm A., Dallmann R., Oettinghaus B., Restelli L.M., Witzig M., Ishihara N., Mihara K., Ripperger J.A., Albrecht U. (2018). Circadian control of DRP1 activity regulates mitochondrial dynamics and bioenergetics. Cell Metab..

[bib78] Schoenhard J.A., Smith L.H., Painter C.A., Eren M., Johnson C.H., Vaughan D.E. (2003). Regulation of the PAI-1 promoter by circadian clock components: differential activation by BMAL1 and BMAL2. J. Mol. Cell Cardiol..

[bib79] Sengupta S., Ince L., Sartor F., Borrmann H., Zhuang X., Naik A., Curtis A., McKeating J.A. (2021). Clocks, viruses, and immunity: lessons for the COVID-19 pandemic. J. Biol. Rhythms.

[bib80] Sengupta S., Tang S.Y., Devine J.C., Anderson S.T., Nayak S., Zhang S.L., Valenzuela A., Fisher D.G., Grant G.R., Lopez C.B., FitzGerald G.A. (2019). Circadian control of lung inflammation in influenza infection. Nat. Commun..

[bib81] Silver A.C., Arjona A., Walker W.E., Fikrig E. (2012). The circadian clock controls toll-like receptor 9-mediated innate and adaptive immunity. Immunity.

[bib82] Solt L.A., Burris T.P. (2012). Action of RORs and their ligands in (patho)physiology. Trends Endocrinol. Metabol. TEM.

[bib83] Solt L.A., Wang Y., Banerjee S., Hughes T., Kojetin D.J., Lundasen T., Shin Y., Liu J., Cameron M.D., Noel R. (2012). Regulation of circadian behaviour and metabolism by synthetic REV-ERB agonists. Nature.

[bib84] Su M., Chen Y., Qi S., Shi D., Feng L., Sun D. (2020). A mini-review on cell cycle regulation of coronavirus infection. Front Vet. Sci..

[bib85] Subramanian A., Tamayo P., Mootha V.K., Mukherjee S., Ebert B.L., Gillette M.A., Paulovich A., Pomeroy S.L., Golub T.R., Lander E.S., Mesirov J.P. (2005). Gene set enrichment analysis: a knowledge-based approach for interpreting genome-wide expression profiles. Proc. Natl. Acad. Sci. U S A.

[bib86] Sutton C.E., Finlay C.M., Raverdeau M., Early J.O., DeCourcey J., Zaslona Z., O'Neill L.A.J., Mills K.H.G., Curtis A.M. (2017). Loss of the molecular clock in myeloid cells exacerbates T cell-mediated CNS autoimmune disease. Nat. Commun..

[bib87] Tegally H., Wilkinson E., Giovanetti M., Iranzadeh A., Fonseca V., Giandhari J., Doolabh D., Pillay S., San E.J., Msomi N. (2020). Emergence and rapid spread of a new severe acute respiratory syndrome-related coronavirus 2 (SARS-CoV-2) lineage with multiple spike mutations in South Africa. MedRxiv.

[bib88] Thakur N., Conceicao C., Isaacs A., Human S., Modhiran N., McLean R.K., Pedrera M., Tan T.K., Rijal P., Townsend A. (2021). Micro-fusion inhibition tests: quantifying antibody neutralization of virus-mediated cell-cell fusion. J. Gen. Virol..

[bib89] Thompson C.P., Grayson N.E., Paton R.S., Bolton J.S., Lourenco J., Penman B.S., Lee L.N., Odon V., Mongkolsapaya J., Chinnakannan S. (2020). Detection of neutralising antibodies to SARS-CoV-2 to determine population exposure in Scottish blood donors between March and May 2020. Euro Surveill..

[bib90] Tipnis S.R., Hooper N.M., Hyde R., Karran E., Christie G., Turner A.J. (2000). A human homolog of angiotensin-converting enzyme. Cloning and functional expression as a captopril-insensitive carboxypeptidase. J. Biol. Chem..

[bib91] Trottein F., Sokol H. (2020). Potential causes and consequences of gastrointestinal disorders during a SARS-CoV-2 infection. Cell Rep..

[bib92] Trump R.P., Bresciani S., Cooper A.W., Tellam J.P., Wojno J., Blaikley J., Orband-Miller L.A., Kashatus J.A., Boudjelal M., Dawson H.C. (2013). Optimized chemical probes for REV-ERBalpha. J. Med. Chem..

[bib93] Volz E., Mishra S., Chand M., Barrett J.C., Johnson R., Geidelberg L., Hinsley W.R., Laydon D.J., Dabrera G., O'Toole A. (2021). Assessing transmissibility of SARS-CoV-2 lineage B.1.1.7 in England. Nature.

[bib94] Vyas M.V., Garg A.X., Iansavichus A.V., Costella J., Donner A., Laugsand L.E., Janszky I., Mrkobrada M., Parraga G., Hackam D.G. (2012). Shift work and vascular events: systematic review and meta-analysis. BMJ.

[bib95] Wan Y., Shang J., Graham R., Baric R.S., Li F. (2020). Receptor recognition by the novel coronavirus from Wuhan: an analysis based on decade-long structural studies of SARS coronavirus. J. Virol..

[bib96] Wang S., Li F., Lin Y., Wu B. (2020). Targeting REV-ERBalpha for therapeutic purposes: promises and challenges. Theranostics.

[bib97] Wang S., Li W., Hui H., Tiwari S.K., Zhang Q., Croker B.A., Rawlings S., Smith D., Carlin A.F., Rana T.M. (2020). Cholesterol 25-Hydroxylase inhibits SARS-CoV-2 and other coronaviruses by depleting membrane cholesterol. EMBO J..

[bib98] Washington N.L., Gangavarapu K., Zeller M., Bolze A., Cirulli E.T., Schiabor Barrett K.M., Larsen B.B., Anderson C., White S., Cassens T. (2021). Emergence and rapid transmission of SARS-CoV-2 B.1.1.7 in the United States. Cell.

[bib99] Widiasta A., Sribudiani Y., Nugrahapraja H., Hilmanto D., Sekarwana N., Rachmadi D. (2020). Potential role of ACE2-related microRNAs in COVID-19-associated nephropathy. Non-coding RNA Res..

[bib100] Wing P.A.C., Keeley T.P., Zhuang X., Lee J.Y., Prange-Barczynska M., Tsukuda S., Morgan S.B., Harding A.C., Argles I.L.A., Kurlekar S. (2021). Hypoxic and pharmacological activation of HIF inhibits SARS-CoV-2 infection of lung epithelial cells. Cell Rep..

[bib101] Wu Y., Tang D., Liu N., Xiong W., Huang H., Li Y., Ma Z., Zhao H., Chen P., Qi X., Zhang E.E. (2017). Reciprocal regulation between the circadian clock and hypoxia signaling at the genome level in mammals. Cell Metab..

[bib102] Xu Z., Shi L., Wang Y., Zhang J., Huang L., Zhang C., Liu S., Zhao P., Liu H., Zhu L. (2020). Pathological findings of COVID-19 associated with acute respiratory distress syndrome. The Lancet. Respirat. Med..

[bib103] Yang L., Wang J., Hui P., Yarovinsky T.O., Badeti S., Pham K., Liu C. (2021). Potential role of IFN-alpha in COVID-19 patients and its underlying treatment options. Appl. Microbiol. Biotechnol..

[bib104] Yang Y., Li N., Qiu J., Ge H., Qin X. (2020). Identification of the repressive domain of the negative circadian clock component CHRONO. Int. J. Mol. Sci..

[bib105] Zhang Q., Bastard P., Liu Z., Le Pen J., Moncada-Velez M., Chen J., Ogishi M., Sabli I.K.D., Hodeib S., Korol C. (2020). Inborn errors of type I IFN immunity in patients with life-threatening COVID-19. Science.

[bib106] Zhang Z., Hunter L., Wu G., Maidstone R., Mizoro Y., Vonslow R., Fife M., Hopwood T., Begley N., Saer B. (2019). Genome-wide effect of pulmonary airway epithelial cell-specific Bmal1 deletion. FASEB J. Off. Publ. Fed. Am. Societies Exp. Biol..

[bib107] Zhao Y., Zhao Z., Wang Y., Zhou Y., Ma Y., Zuo W. (2020). Single-cell RNA expression profiling of ACE2, the receptor of SARS-CoV-2. Am. J. Respir. Crit. Care Med..

[bib108] Zhou D., Duyvesteyn H.M.E., Chen C.P., Huang C.G., Chen T.H., Shih S.R., Lin Y.C., Cheng C.Y., Cheng S.H., Huang Y.C. (2020). Structural basis for the neutralization of SARS-CoV-2 by an antibody from a convalescent patient. Nat. Struct. Mol. Biol..

[bib109] Zhuang X., Magri A., Hill M., Lai A.G., Kumar A., Rambhatla S.B., Donald C.L., Lopez-Clavijo A.F., Rudge S., Pinnick K. (2019). The circadian clock components BMAL1 and REV-ERBalpha regulate flavivirus replication. Nat. Commun..

