# Supplementary material for: The circadian clock component BMAL1 regulates SARS-CoV-2 entry and replication in lung epithelial cells
Source: iScience. 2021 Sep 16;24(10):103144. doi: 10.1016/j.isci.2021.103144 (PMC8443536; doi:10.1016/j.isci.2021.103144)
Supplement: Document S1. Figures S1–S5 and Table S1 [file mmc1.pdf]

iScience, Volume *24*

Supplemental information

The circadian clock component BMAL1 regulates SARS-CoV-2 entry and replication in lung epithelial cells

Xiaodong Zhuang, Senko Tsukuda, Florian Wrensch, Peter A.C. Wing, Mirjam Schilling, James M. Harris, Helene Borrmann, Sophie B. Morgan, Jennifer L. Cane, Laurent Mailly, Nazia Thakur, Carina Conceicao, Harshmeena Sanghani, Laura Heydmann, Charlotte Bach, Anna Ashton, Steven Walsh, Tiong Kit Tan, Lisa Schimanski, Kuan-Ying A. Huang, Catherine Schuster, Koichi Watashi, Timothy S.C. Hinks, Aarti Jagannath, Sridhar R. Vausdevan, Dalan Bailey, Thomas F. Baumert, and Jane A. McKeating

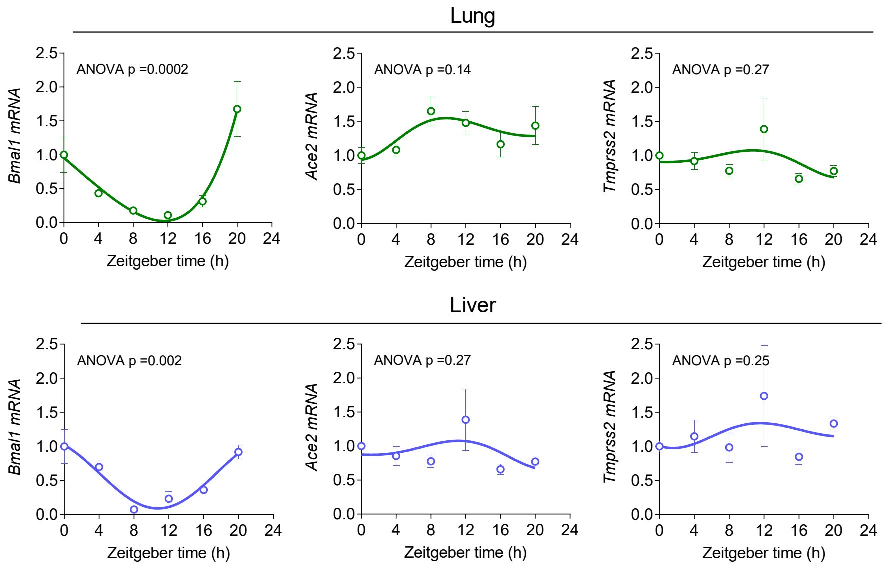

Figure S1. *Bmal1, Ace2 and Tmprss2* transcripts in mouse tissues.
Lung and liver tissues from circadian entrained mouse were harvested at 4h intervals and *Bmal1, Ace2* and *Tmprss2* mRNAs quantified by qPCR and expressed relative to CT0. Data represent the mean ± S.E.M., n = 5, Kruskal–Wallis ANOVA with Dunn's test. (**Related to Fig.1**).

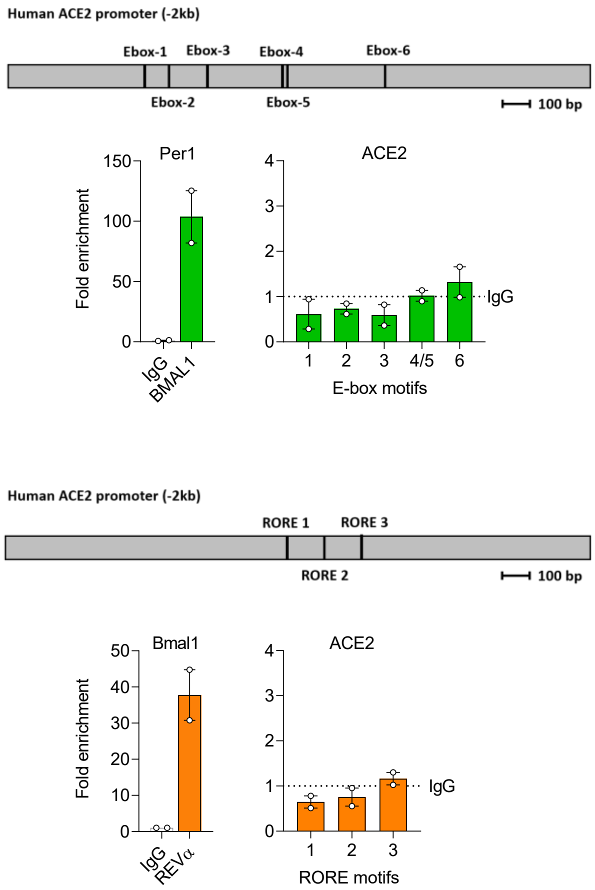

Figure S2. ChIP analysis of BMAL1 or REV-ERBα binding ACE2 promoter in Calu-3 cells.
Chromatin extracts from Calu-3 cells were immunoprecipitated using antibodies specific for BMAL1, REV-ERBα or rabbit IgG. qRT-PCR for ACE2 promoter DNA using primers over defined Ebox and RORE motifs or control host gene Per1 for BMAL1 ChIP and Bmal1 for REV-ERBα (positive control) was performed. IP data is presented relative to the rabbit IgG control shown as the dotted line (mean $\pm$ S.E.M., n = 2, Kruskal–Wallis ANOVA with Dunn's test). (**Related to Fig.1**).

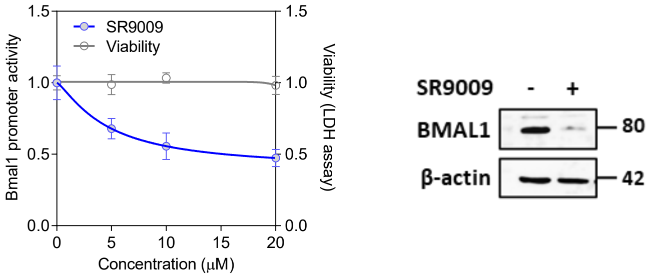

Figure S3. Effect of REV-ERB agonist SR9009 on BMAL promoter activity and expression.
Calu-3 cells stably expressing a Bmal1 promoter driven luciferase reporter were treated with SR9009 for 24h and promoter activity quantified by measuring luciferase activity. Cytotoxicity was determined using an LDH assay. Data are expressed relative to untreated cells and represent the mean±S.E.M., n = 16. Calu-3 cells were treated with SR9009 (20 μM) for 24h and assessed for BMAL1 expression together with housekeeping β-actin by western blotting. **(Related to Fig.1).**

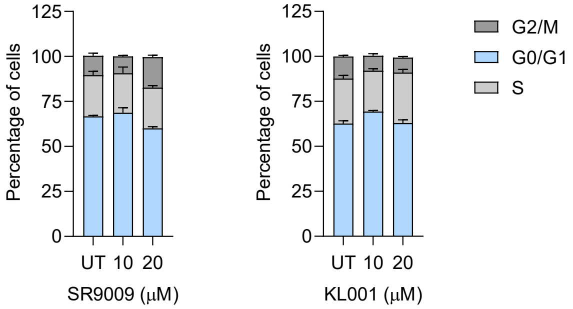

Figure S4 Cell cycle analysis of Calu-3 cells.

Calu-3 cells were seeded at 8 x 10^5/well of a 6-well plate and cell cycle analysis performed at 48h post SR9009 or KL001 treatment. The proportion of cells per phase was determined by detecting BrdU incorporation and Propidium Iodide staining by flow cytometry. Statistics were tested with Two-way ANOVA (n = 3 per time point) and revealed no significant differences in cell cycle phase across time points**. (Related to Fig.3)**

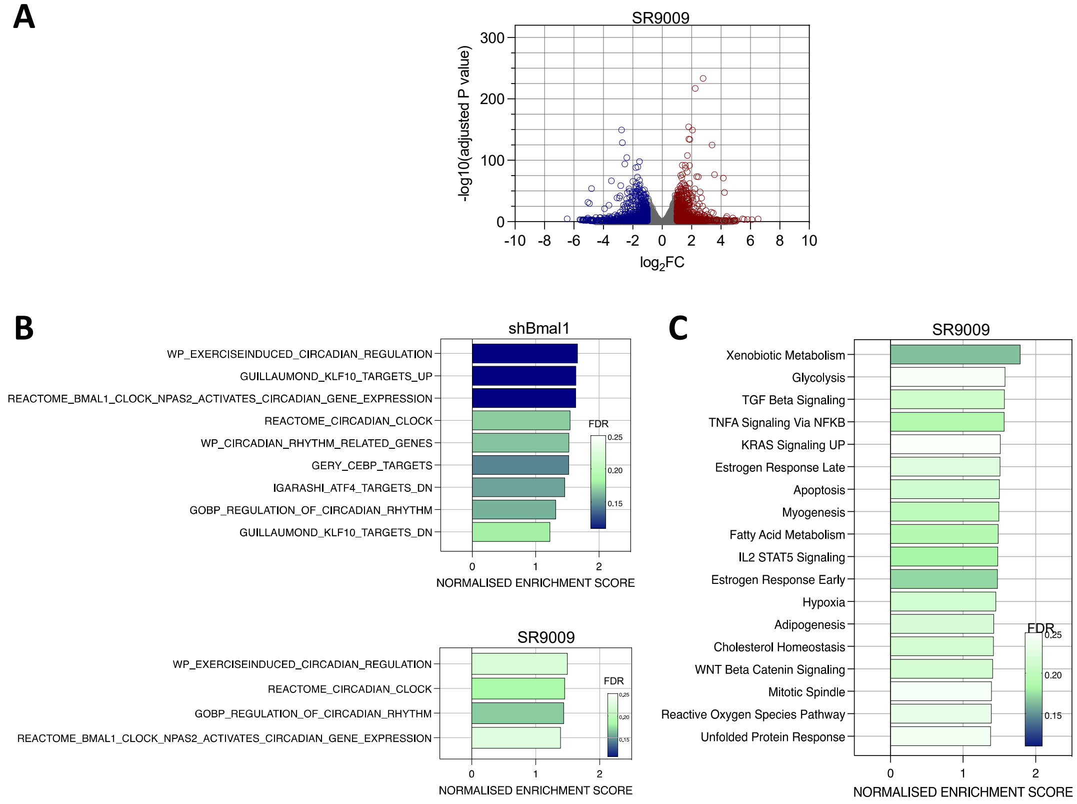

Figure S5. RNA-seq analysis of Bmal1 silenced and SR9009 treated Calu-3 cells.

(**A**) Differentially expressed genes in SR9009 treated Calu3 cells. Calu-3 cells were treated with SR9009 (20 µM) for 24h and gene expression quantified by RNA sequencing. Differential expression analysis was performed using DESeq2 Package between SR9009 and untreated cells. Volcano plot shows significantly differentially expressed genes based on a log2FC of +/- 1 and an adjusted (Benjamini Hochberg) P value of 0.05. Red points denote significant upregulation, blue denotes downregulation. (**B**) Validation of RNA-seq data on previously reported clock genes. All Gene sets from MSigDB were searched for the term “Circadian”, and 24 gene sets obtained. GSEA was performed between shBmal1 and SR9009 against untreated cells. Gene sets were filtered for size based on >15 and <500 genes, and for their expression in both experimental groups. 9/12 circadian gene sets were significantly enriched in shBmal1, and 4/13 SR9009 treated cells. Gene sets are ranked by NES and coloured by FDR. (**C**) Gene set enrichment analysis (GSEA) for SR9009 regulated host pathways. Using the Hallmarks gene sets from the molecular signatures database, 18 out of 50 gene sets were significantly upregulated in SR9009 above untreated cells, at an FDR of less than 25%. Significantly enriched hallmarks were plotted, ranked by normalised enrichment score, and coloured by FDR. (**Related to Fig.4**).

Table S1. Oligonucleotides, related to STAR methods.

qPCR Primers (5’-3’)
Human ACE2 forward: GGGATCAGAGATCGGAAGAAGAAA
Human ACE2 reverse: AGGAGGTCTGAACATCATCAGTG
Human TMPRSS2 forward: AGGTGAAAGCGGGTGTGAGG
Human TMPRSS2 reverse: ATAGCTGGTGGTGACCCTGAG
Human B2M forward: CTACACTGAATTCACCCCCACTG
Human B2M reverse: ACCTCCATGATGCTGCTTACATG
Human BMAL1: TaqMan Gene Expression Assay, ThermoFisher, Hs00154147
Mouse Ace2 forward: AATTCCACTGAAGCTGGGCA
Mouse Ace2 reverse: CCATTCAGTGTTCCACCCCA
Mouse Tmprss2 forward: CTCAGGCAACGTTGACC
Mouse Tmprss2 reverse: GTCTCCTGGTGAGGCATTCA
Mouse Bmal1 forward: AAGTGCAACAGGCCTTCAGT
Mouse Bmal1 reverse: GGTGGCCAGCTTTTCAAATA
Mouse GAPDH: TaqMan Gene Expression Assay, ThermoFisher, Hs99999905_m1
SARS-CoV-2_N forward: CACATTGGCACCCGCAATC
SARS-CoV-2_N reverse: GAGGAACGAGAAGAGGCTTG
APOL3 forward: CCAGTGCATCTGCAAATCCTGC
APOL3 reverse: CTTTCTGCTGCACATATTCGTCC
APOL6 forward: CCACCTCTACTGCTGTCATCTC
APOL6 reverse: GAGCGTTCCAACGTACCACTCA
ATF3 forward: CGCTGGAATCAGTCACTGTCAG
ATF3 reverse: CTTGTTTCGGCACTTTGCAGCTG
CEACAM1 forward: CACGCCAATAACTCAGTCACTGG
CEACAM1 reverse: TTGTGGAGCAGGTCAGGTTCAC
CH25 reverse: AGGCAGAACAGGATGTGGTGCA
CH25H forward: CAGACCCTCTACCAGCATGTGA
CREB3L3 forward: GAAGCCTCTGTGACCATAGACC
CREB3L3 reverse: GGAGGTCTTTCACGGTGAGATTG
CYP1B1 forward: GCCACTATCACTGACATCTTCGG
CYP1B1 reverse: CACGACCTGATCCAATTCTGCC
FBXO6 forward: TCGGCTGACTACTTCGTGTTGG
FBXO6 reverse: TGCTGGAAGAGGATGTAGCGGA
IFIT1 forward: GCCTTGCTGAAGTGTGGAGGAA
IFIT1 reverse: ATCCAGGCGATAGGCAGAGATC
IFIT2 forward: GGAGCAGATTCTGAGGCTTTGC
IFIT2 reverse: GGATGAGGCTTCCAGACTCCAA
IFIT3 forward: CCTGGAATGCTTACGGCAAGCT
IFIT3 reverse: GAGCATCTGAGAGTCTGCCCAA
IFITM1 forward: GGCTTCATAGCATTCGCCTACTC
IFITM1 reverse: AGATGTTCAGGCACTTGGCGGT
IFITM2 forward: GGCTTCATAGCATTCGCGTACTC
IFITM2 reverse: AGATGTTCAGGCACTTGGCGGT
IFITM3 forward: CTGGGCTTCATAGCATTCGCCT
IFITM3 reverse: AGATGTTCAGGCACTTGGCGGT
IFNB1 forward: CTTGGATTCCTACAAAGAAGCAGC
IFNB1 reverse: TCCTCCTTCTGGAACTGCTGCA

qPCR Primers (5'-3')
IFNL1 forward: AACTGGGAAGGGCTGCCACATT
IFNL1 reverse: GGAAGACAGGAGAGCTGCAACT
ISG15 forward: CTCTGAGCATCCTGGTGAGGAA
ISG15 reverse: AAGGTCAGCCAGAACAGGTCGT
ISG20 forward: ACACGTCCACTGACAGGCTGTT
ISG20 reverse: ATCTTCCACCGAGCTGTGTCCA
Mx2 forward: AAAAGCAGCCCTGTGAGGCATG
Mx2 reverse: GTGATCTCCAGGCTGATGAGCT
OPTN forward: ACTCTGACCAGCAGGCTTACCT
OPTN reverse: CTATGTCAGGCAGAACCTCTCC
RASSF4 forward: CCAATGTGAGGGTCAACAGCAC
RASSF4 reverse: CTCGTGAACGATGTAGAGTGCG
RNF19B forward: AGACACAGCCAGTCTTGGTGCA
RNF19B reverse: GCTGATAGTGGCTTGGTTTGGC
RTP4 forward: GACGCTGAAGTTGGATGGCAAC
RTP4 reverse: GTGGCACAGAATCTGCACTTGG
SAA1 forward: TCGTTCCTTGGCGAGGCTTTTG
SAA1 reverse: AGGTCCCCTTTTGGCAGCATCA
SAMHD1 forward: CTCGCAACTCTTTACACCGTAGA
SAMHD1 reverse: TTTCCTCCAGCACCTGTAATCTC
SLC15A3 forward: GCAAGAGGACATCGCCAACTTC
SLC15A3 reverse: TTGGGATGTGGAGGTGAAGACC
TBP forward: CCCATGACTCCCATGACC
TBP reverse: TTTACAACCAAGATTCACTGTGG
TXNIP forward: CAGCAGTGCAAACAGACTTCGG
TXNIP reverse: CTGAGGAAGCTCAAAGCCGAAC
**ChIP-PCR Primers (5'-3')**
Per1 promoter forward: GTCAAGGAAAATCCCCAGCTTCTG
Per1 promoter reverse: CCAAGATTGGTGACGTAAATGCCA
Bmal1 promoter forward: TTGGGCACAGCGATTGGT
Bmal1 promoter reverse: GTAAACAGGCACCTCCGTCC
ACE2 promoter Ebox1 forward: AGCATCACAAGAAACCTATAGGCA
ACE2 promoter Ebox1 reverse: TGCCTCCACTATTGCTTTTAGAC
ACE2 promoter Ebox2 forward: TGTCCACTGCTTTTCCTTATTCCA
ACE2 promoter Ebox2 reverse: TGCCTATAGGTTTCTTGTGATGCT
ACE2 promoter Ebox3 forward: TGGCAGGGTTCAAGGGCCTACC
ACE2 promoter Ebox3 reverse: TGGAATAAGGAAAAGCAGTGGACA
ACE2 promoter Ebox4/5 forward: GGCTACAGAGGATCAGGAGTTGACA
ACE2 promoter Ebox4/5 reverse: TGCCTCCTTCTCCTTCACTTACCT
ACE2 promoter Ebox6 forward: AACTTAGCTGGGCGTGGTGGTG
ACE2 promoter Ebox6 reverse: CGCGATCTCGGCTCACTGCAAG
ACE2 promoter RORE1 forward: GGCTACAGAGGATCAGGAGTTGACA
ACE2 promoter RORE1 reverse: TGCCTCCTTCTCCTTCACTTACCT
ACE2 promoter RORE2 forward: TGCAAAGGCAGATCAGGAGAGT
ACE2 promoter RORE2 reverse: GCCACTGTGCCCAGCCATTTCT
ACE2 promoter RORE3 forward: GCACTTTGGGAGGCCGAGTTGG
ACE2 promoter RORE3 reverse: CACCACCACGCCCAGCTAAGTT
